# Current Scientific Advances in Vaccines Against UTIs: Challenges and Prospects

**DOI:** 10.3390/microorganisms13122714

**Published:** 2025-11-28

**Authors:** Baoying Wang, Yuhui Wang, Haodi Liu, Mingyang Yu, Shuaishuai Wang, Lele Liu, Hailong Wang, Daizhou Zhang, Haining Tan

**Affiliations:** 1 National Glycoengineering Research Center, Shandong University, Qingdao 266237, China; wbygoodbye@163.com (B.W.); wangyuhui@edu.cn (Y.W.); liuhaodi1210@163.com (H.L.); swang1@sdu.edu.cn (S.W.); 2 NMPA Key Laboratory for Quality Research and Evaluation of Carbohydrate-Based Medicine, Shandong University, Qingdao 266237, China; 3 Shandong Provincial Technology Innovation Center of Carbohydrate, Shandong University, Qingdao 266237, China; 4 School of Pharmaceutical Sciences, Shandong University, Jinan 250012, China; 5 School of Life Sciences, Shandong University, Qingdao 266237, China; 202300141057@mail.sdu.edu.cn (M.Y.); liulele@sdu.edu.cn (L.L.); 6 State Key Laboratory of Microbial Technology, Shandong University, Qingdao 266237, China; wanghailong@sdu.edu.cn; 7 Shandong Academy of Pharmaceutical Sciences, Jinan 250101, China

**Keywords:** urinary tract infection, uropathogenic *Escherichia coli*, antibiotic resistance, polysaccharide conjugate vaccines, biosynthesis

## Abstract

Urinary Tract Infection (UTI), the second most common infectious disease globally, poses a particularly significant threat to adult female populations. Epidemiological data show that Uropathogenic *Escherichia coli* (UPEC) is responsible for approximately 75% to 90% of UTI cases. Currently, antibiotic therapy constitutes the primary treatment for UTIs. However, the rising prevalence of antimicrobial resistance, particularly among *Escherichia coli* strains, is increasingly compromising treatment efficacy and elevating the risk of therapeutic failure and complications. Considering this serious challenge, the urgent exploration and development of alternative therapies for UTIs, particularly vaccine therapies, to supplement or replace antibiotic use is crucial. Polysaccharide conjugate vaccines represent a highly successful strategy in bacterial vaccine development, playing a pivotal role in the prevention and control of human infectious diseases. This article aims to review the research progress on UTI vaccines and focus on the preparation methods of polysaccharide conjugate vaccines, encompassing traditional chemical conjugation techniques and emerging biosynthetic methods. Through an in-depth analysis of biosynthetic methods, this article identifies the key steps and proposes insights for further optimization strategies for polysaccharide conjugate vaccines. It is hoped that this study will provide a more comprehensive and in-depth reference for the development of UTI vaccines.

## 1. Introduction

Urinary tract infection (UTI) is an inflammatory condition typically caused by bacterial infection, beginning in the urethra and often ascending to the bladder or kidneys. *Escherichia coli* (*E. coli*) is the most common pathogen, although other bacteria, such as *Klebsiella* species, can also cause infections [1,2,3]. The urinary tract frequently serves as a source of infection in young children and infants, and it is the most prevalent bacterial infection among children under 2 years old, both in community and hospital settings [4,5,6,7,8,9], impacting approximately 1.7% of male children and 8.4% of female children before they reach the age of seven [10]. Notably, UTIs primarily occur in sexually active young women and the female-to-male ratio of UTI is significantly skewed, with women accounting for approximately 81% of all reported cases [11,12,13,14]. Other groups at elevated risk include the elderly and individuals undergoing catheterization procedures, who may suffer from more severe complications due to delayed diagnosis or treatment. Furthermore, UTIs demonstrate a high prevalence rate and patients frequently experience recurrent episodes, thereby imposing a substantial clinical and economic burden on healthcare systems. Recurrent urinary tract infections (rUTIs) are clinically defined as either two episodes of acute bacterial cystitis within six months or three culture-proven uncomplicated acute cystitis episodes within one year, with confirmed symptom resolution and negative cultures between episodes. It is now established that rUTIs develop through two distinct pathogenic pathways: bacterial reinfection from exogenous sources, and bacterial persistence due to the formation of intracellular reservoirs [15,16]. Seminal studies have demonstrated that UPEC invades the superficial umbrella cells of the bladder epithelium and evades antibiotic clearance through the establishment of quiescent intracellular reservoirs (QIRs). These QIRs can subsequently reactivate, leading to recurrent symptomatic episodes caused by the same bacterial strain. The financial burden of UTIs in the inpatient setting ranges from €5700 per case in Europe to US$13,000 per case in the USA, resulting in an estimated annual expenditure of over US$6 billion in the USA alone [3,14,17,18]. Although current epidemiological data likely underestimate the true burden of UTIs, their substantial impact on both individual health and societal healthcare systems remains unequivocally evident.

UTIs can be categorized as uncomplicated or complicated UTIs, depending on the physical condition of the host. UPEC is the most common pathogen for both uncomplicated and complicated UTI, making up 75% and 65% of infections, respectively [3,19]. Uncomplicated urinary tract infections (UTIs) are typically diagnosed in otherwise healthy individuals without significant comorbidities and generally present with mild clinical manifestations that respond well to first-line antibiotic therapy. Complicated UTIs predominantly occur in individuals with underlying conditions such as anatomical abnormalities, diabetes, or immunosuppression. These factors increase treatment challenges, necessitating more aggressive management to prevent complications like renal damage or sepsis [13]. Beyond this clinical categorization, UTIs are also traditionally classified as upper and lower UTIs, based on the anatomical site of infection, which is crucial for guiding appropriate diagnosis and treatment. Similarly, the distribution differs between upper and lower UTIs, with UPEC causing 70–80% of pyelonephritis cases compared to 80–90% of cystitis episodes [3,20]. Lower UTIs usually affect the urethra and bladder and may be accompanied by frequent urination, suprapubic discomfort, or significant hematuria. In contrast, upper UTIs, which involve the kidneys and ureters, are typically systemic, presenting with symptoms such as fever (≥37.8 °C), flank pain, chills, nausea, and vomiting. In contrast, fever is relatively uncommon in lower UTIs; when it does occur, it usually indicates a more complicated infection. However, it is essential to acknowledge that not all UTI patients exhibit these classic symptoms. Therefore, in addition to clinical evaluation, physiological and biochemical indicators are frequently measured to assist in diagnosis. A study by Rajanbir Kaur and Rajinder Kaur lists the terms used to describe various conditions associated with UTIs, providing a valuable reference for clinicians and researchers [12]. Additionally, European experts have proposed the ORENUC classification system under the EAU/ESIU framework, which defines distinct severity groups for UTIs based on clinical manifestations, categorization of risk factors, and the availability of appropriate antimicrobial treatment [21,22].

**Figure 1:**
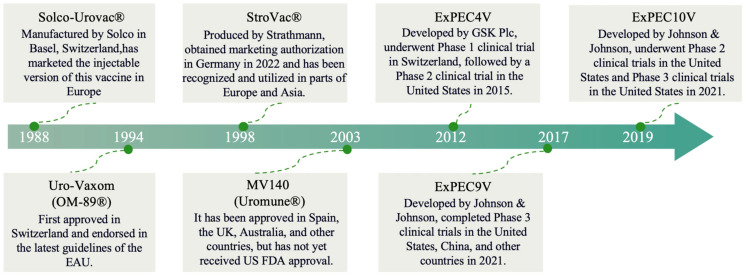
The progression and advancements in vaccine technologies.

Since the advent of sulfonamides in the 1940s, antimicrobial agents, particularly antibiotics, have been employed as the primary means of addressing UTIs [23]. Nitrofurantoin experienced a significant decline in usage during the late 1970s following the introduction of newer antibiotics. However, due to the increasing resistance to fluoroquinolones and other broad-spectrum antimicrobials, nitrofurantoin—an older antibiotic with a low propensity for resistance development—has re-emerged as a first-line treatment for uncomplicated UTIs [24]. UTIs are the second most common indication for antibiotic prescription, trailing only respiratory infections in terms of frequency. This widespread use of antibiotics for UTI treatment underscores the significant burden of these infections on healthcare systems and the importance of effective therapeutic strategies. In the context of first-line therapeutic interventions, four agents are prominently recommended: nitrofurantoin, trimethoprim-sulfamethoxazole (TMP-SMX), pivmecillinam, and fosfomycin tromethamine [25,26,27]. Despite its advantages, prolonged administration can lead to severe hepatotoxicity and pulmonary toxicity, necessitating careful clinical monitoring [27]. Furthermore, nitrofurantoin exhibits limited efficacy in high-risk populations, such as pediatric and female patients with recurrent or complicated UTIs. Fosfomycin has low baseline resistance, but substantial clinical usage and demonstrated propensity for in vitro/in vivo resistance mutations constrain susceptibility rates to approximately 5–10% [28,29]. It is estimated that 30 to 60% of antibiotic prescriptions for UTIs prescriptions may not be appropriate according to current clinical guidelines, only 34% of outpatient antibiotic prescriptions for cystitis are given with the correct antibiotic, dose, and duration [30,31,32]. Multiple evidence-based guidelines formulated by prestigious international societies, such as those from the Infectious Diseases Society of America (IDSA) [33], the American Urological Association (AUA) [34], the American Academy of Family Physicians (AAFP) [35], and the American Urogynecologic Society (AUGS) [36], have delineated comprehensive pathways for the assessment and management of UTIs [23,37,38,39]. However, current clinical practice appears to deviate significantly. Multiple studies document poor adherence to major guidelines like those from IDSA, primarily through the overuse of fluoroquinolones, extended therapy duration, and underuse of first-line agents for uncomplicated UTIs [40,41,42]. These deviations, stemming from factors like diagnostic uncertainty and habitual prescribing, contribute significantly to the development of antimicrobial resistance (AMR), impacting public health and healthcare systems. It reduces treatment effectiveness, burdens healthcare, and promotes multidrug resistance, complicating disease management globally [43,44]. Vaccination offers a proactive approach to mitigate antibiotic dependence in recurrent UTIs, serving as a complementary strategy to antimicrobial stewardship [45]. This is especially valuable in clinical settings where adherence to treatment guidelines remains inconsistent, thereby helping to circumvent variability in antibiotic prescribing practices. Vaccine development should be considered as part of a multifaceted approach to UTI management, working synergistically with antimicrobial stewardship programs while addressing the limitations of current antibiotic therapies. Figure 1 summarizes the most important steps in the history of vaccines targeting UTIs.

However, the paradigm shift in understanding rUTIs carries profound implications for vaccine design. A vaccine that only elicits antibodies against surface adhesins may prevent initial bacterial colonization and reinfection but is likely insufficient to eliminate established reservoirs or target bacteria sheltered within host cells. Consequently, an ideal UTI vaccine should stimulate not only humoral immunity to block bacterial adhesion but also robust cellular immune responses that are mediated primarily by tissue-resident memory T cells and are capable of recognizing and eliminating infected bladder cells to eradicate these persistent bacterial reservoirs. Given these complex requirements, in this review, we critically assess the therapeutic potential of vaccination strategies against UTIs within a global public health framework, synthesizing current scientific advances, identifying enduring translational barriers, and delineating key determinants for next-generation vaccine design. Finally, we offer a forward-looking perspective, fervently hoping that this paper will serve as a catalyst, directing the scientific community’s attention to the latest breakthroughs in the field of UTI therapeutics.

## 2. Pathogenesis of UTI

Uropathogenic bacteria (such as *E. coli*, *Klebsiella pneumoniae*, *Staphylococcus aureus*, etc.) cause UTIs through a coordinated sequence of virulence mechanisms [46,47,48]. The infection begins with bacterial colonization of the urothelial cells lining the urethra and bladder, a process mediated by specific bacterial adhesins such as type 1 pili in UPEC [49]. The FimH adhesin located at the pilus tip recognizes and binds with high affinity to host receptors such as uroplakin Ia (UPIa), enabling firm attachment and resistance to clearance by urinary flow [50]. Following adhesion, bacteria are internalized into host cells via receptor-mediated endocytosis and form intracellular bacterial communities (IBCs) [46]. Within the intracellular compartment, pathogens such as UPEC secrete effector proteins including the phospholipase PldA, which disrupts the endosomal membrane, facilitating bacterial escape into the cytoplasm where they proliferate rapidly and establish new IBCs [51]. This intracellular niche provides a unique immune-privileged sanctuary that shields bacteria from host defenses. Furthermore, pathogens enhance their antibiotic resistance through biofilm formation [52]. It is important to distinguish IBCs—transient structures formed within living host cells—from classical biofilms, which typically develop on abiotic surfaces such as catheters or on necrotic tissues; both configurations significantly contribute to chronic and recurrent infections. Bacterial persistence is further promoted by multiple immune evasion strategies, such as modulating host cell signaling pathways to suppress epithelial exfoliation and delay apoptosis, thereby maintaining a protected intracellular reservoir [53]. If the infection remains unresolved, pathogens can ascend via the ureters to colonize the kidneys, resulting in pyelonephritis and, in severe cases, progression to bacteremia or sepsis [54].

The interconnected nature of these pathogenic mechanisms creates significant therapeutic challenges in UTI management. Contemporary investigations highlight the crucial need to decipher the molecular mechanisms underlying bacterial colonization, tissue invasion, immune evasion, and biofilm formation to inform next-generation therapeutic development.

### 2.1. UPEC Infection Mechanisms

*E. coli* strains that cause UTIs are also known as UPEC. An analysis of epidemiological data on UTIs reveals that UPEC constitutes the primary etiological agent, accounting for approximately 75% of uncomplicated UTI cases [3,55]. UPEC strains are distributed among 45 O-serogroups, with 64.0% concentrated in 12 predominant serogroups: O1, O2, O4, O6, O7, O14, O15, O18, O21, O25, O75, and O175. These strains commonly harbor UPEC virulence genes, including *pap* (45.8%), *hly* (44.0%), *aer* (39.6%), *sfa* (29.8%), and *cnf* (23.6%) [56]. UPEC establishes colonization within the bladder through a variety of virulence determinants, which play a pivotal role in the pathogenesis of UTIs. As UPEC represents the predominant causative pathogen of UTIs, comprehensive investigation of its virulence mechanisms and development of optimized prevention and treatment approaches are critical. The diverse virulence profiles of clinical UPEC isolates have informed the composition of both whole-cell and subunit vaccines, as detailed in Table 1 and Table 2.

**Figure 2:**
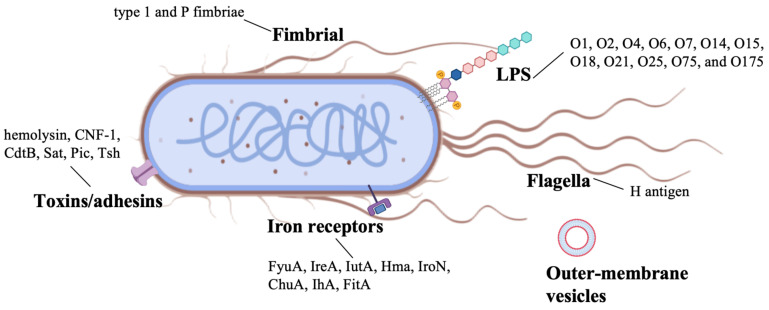
Virulence factors of uropathogenic *Escherichia coli* strains. The figure was created in BioRender. Baoying Wang (2025). https://BioRender.com/5kdw4yz (accessed on 20 November 2025).

UPEC exploits a multifaceted repertoire of adhesive factors to facilitate colonization within the urinary tract, manipulate host physiological processes and immune defenses, and establish persistent residence in host tissues (Figure 2). These factors have been studied mostly in Gram-negative uropathogens and can be divided into two categories: pili and non-pili adhesins. The diverse pili and adhesins present on the UPEC surface play crucial roles in mediating adhesion and colonization. Adhesins, a diverse array of bacterial surface-assembled adhesive proteins, have emerged as important investigative targets, particularly the adhesive fibers known as pili. Pili are elongated fibers protruding from the bacterial envelope, with only two types (type 1 and P pili) being closely associated with UTI pathogenesis [81,82,83]. FimH, serving as the tip adhesin of type 1 pili, comprises an N-terminal lectin domain and a C-terminal fibronectin domain, functioning as a pathogen-associated molecular pattern (PAMP) [84,85]. Upon attachment to superficial receptors of uroepithelial cells, it triggers inflammatory responses, manifesting as painful symptoms such as dysuria. P pili are capable of binding to α-D-galactopyranosyl-1,4-β-D-galactopyranoside within globoseries glycolipids [86], with Pap G serving as its tip adhesin. The majority of UPEC adherence to uroepithelial cells is mediated by P pili [87]. Additional adhesins, such as TosA, are released via a type I secretion pathway and are present in approximately 30% of urinary isolates [88,89]. In murine UTI models, the iron-regulated adhesin Iha facilitates adhesion to bladder cells, enhancing UPEC’s advantage [90]. UPEC also produces three key toxins—hemolysin, CNF1, and secreted autotransporter toxins—that aid in colonization of the ureters and kidneys [91]. Iron acquisition, crucial for bacterial virulence, is mediated by the siderophore uptake system, including TonB-dependent receptors [92,93,94]. Recently, the intimin-/invasin-like fimbria adhesins were also identified in UPEC, but their biogenesis and activity are still largely unexplored [3,95]. As UPEC proliferates, it forms biofilms, which are essential for urinary tract colonization and infection initiation.

Lipopolysaccharide (LPS), a major component of the outer membrane in Gram-negative bacteria like UPEC, serves as both a critical virulence factor and a promising vaccine target. Structurally composed of O-antigen, core oligosaccharide, and lipid A, LPS mediates host-pathogen interactions through multiple mechanisms [96,97]: the highly variable O-antigen facilitates immune evasion, while the conserved core region and lipid A trigger proinflammatory responses via TLR4 signaling. As a vaccine target, LPS offers distinct advantages: its surface-exposed epitopes are accessible to antibodies. Targeting the conserved core oligosaccharide is a strategy for achieving broad-spectrum protection, whereas immunodominant O-antigens of prevalent serotypes (e.g., O1, O6) are key for type-specific immunity (Figure 2). Preclinical studies demonstrate that LPS-based vaccines can elicit protective antibodies that neutralize UPEC adhesion, block biofilm formation, and enhance bacterial clearance [98,99]. Their immunogenicity is typically enhanced through conjugation to carrier proteins, overcoming the T-cell-independent nature of pure polysaccharide antigens [100]. Despite challenges posed by O-antigen diversity, recent advances in glycoconjugate technology have reinvigorated interest in LPS as a viable target for developing vaccines against recurrent UTIs [101,102,103]. The immunodominant O-antigen polysaccharides of LPS (shown in Figure 2) represent prime targets for conjugate vaccine development.

### 2.2. Virulence Factors of Other Uropathogens

Other uropathogens, including Gram-negative bacteria such as *Klebsiella pneumoniae*, *Pseudomonas aeruginosa*, and *Proteus mirabilis*, as well as Gram-positive bacteria such as *Enterococcus faecalis*, *Enterococcus faecium*, *Streptococcus agalactiae* (also known as Group B *Streptococcus* or GBS), *Staphylococcus aureus*, and *Staphylococcus saprophyticus*, are becoming increasingly common in older individuals and those with comorbidities or hospital-acquired infections [1,2]. Therefore, in-depth exploration of the pathogenic mechanisms of these bacteria is crucial for designing effective anti-adhesion strategies to combat infections caused by these pathogens. Typically, like UPEC, these uropathogens also colonize, disrupt the host’s physiology and immune responses, and persist in tissues through pili and non-pili adhesins. Non-chaperone-usher pili (non-CUP), involved in adhesion during UTI, include amyloid fibres, such as UPEC curli, with possible roles also attributed to *P. aeruginosa* Fap and *Enterococci Esp* fibres [81,104,105]. Another common non-CUP in Gram-negative uropathogens is the type IV pilus (T4P), which has been extensively characterized in *Pseudomonas aeruginosa* and is also a critical virulence factor for prevalent uropathogens such as *Acinetobacter baumannii* and specific pathovars of *Escherichia coli*. These are unique dynamic pili, with rapid cycles of extension and retraction after polymerization and depolymerization of pilin filaments.

**Figure 3:**
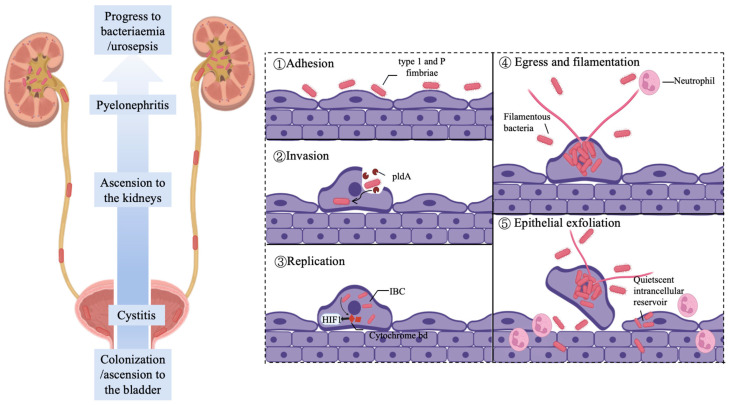
Uropathogen adhesion during UTI progression. UPEC ascends through the urethra and ureters to establish bladder infection. The pathogenesis of urinary tract infections involves UPEC attaching to bladder epithelial cells via type 1 pili. After cellular entry, bacteria use PldA phospholipase to escape endosomes and multiply in the cytoplasm, forming bacterial clusters. UPEC stabilizes HIF1 protein through cytochrome bd oxidase to inhibit cell death. Upon host cell rupture, filamentous UPEC resists neutrophil clearance. Subsequent exfoliation of superficial cells allows bacterial penetration into deeper tissues, establishing latent infection foci that cause recurrent infections. The figure was created in BioRender. Baoying Wang (2025). https://BioRender.com/dnip437 (accessed on 20 November 2025).

Uropathogens also display non-pili adhesins for colonization, including the Dra/Afa family and autotransporter (AT) adhesins. Dra/Afa adhesins typically require five elements to maintain their biosynthesis and function—transcriptional regulators, a periplasmic chaperone, and anchoring, invasin, and adhesin proteins. AT adhesins are usually produced by type V secretion systems and have been described in UPEC and *P. mirabilis*. Although the functions of AT adhesins can vary significantly from adhesins to toxins, they generally share three structural regions [95,106,107]: (1) an N-terminal signal peptide; (2) a passenger domain, which can either be anchored to the cell envelope or released into the extracellular environment; and (3) a translocation domain embedded in the outer membrane. For uropathogenic Gram-positive cocci (mainly *E. faecalis*, *S. saprophyticus*, and *S. aureus*), non-fimbrial adhesins include microbial surface components recognizing adhesive matrix molecules (MSCRAMMs). These proteins are cell-wall-anchored and often have two tandemly arranged immunoglobulin G-like folded domains involved in protein binding. There are also anchorless adhesins, which lack a traditional cell-wall anchor or retention motif, as well as other *S. saprophyticus*-specific adhesins (such as Uaf and Aas) (Figure 3).

Adhesins and pili play pivotal roles in the urinary tract colonization and persistence of uropathogens—critical initial steps in the infection cascade. These surface structures mediate bacterial attachment to host cells, a prerequisite for subsequent virulence mechanisms. Beyond adhesins and pili, additional virulence factors—including toxins (e.g., hemolysin and CNF1), iron acquisition systems, urease, and biofilm formation—contribute significantly to uropathogen pathogenicity. Urease, particularly prominent in *Proteus mirabilis* and some *Klebsiella* species, hydrolyzes urea to ammonia, leading to urine alkalinization and the formation of struvite crystals. This not only facilitates stone formation and tissue damage but also promotes bacterial persistence and biofilm development within the urinary tract [108,109,110,111]. These elements facilitate immune evasion, tissue damage, and chronic infection, and their mechanisms are elaborated as potential therapeutic targets in Table 3.

## 3. Advances in UTI Vaccine Development

### 3.1. Pathogen-Specific Vaccine Strategies

Current evidence from clinical development demonstrates that whole-cell vaccines—encompassing both attenuated vaccines and inactivated whole-cell formulations-exhibit superior protective efficacy compared to subunit or purified antigen vaccines, attributable to their preservation of the complete pathogen antigenic repertoire. Inactivated vaccines, such as Uro-Vaxom (OM-89) and Solco-Urovac, leverage complete bacterial antigens to elicit comprehensive immune responses that may provide broad protection against multiple strains. Table 1 serves as a preliminary overview, providing insight into the contemporary landscape of vaccine development targeted at UTIs. Currently, there are three major types of whole-cell inactivated vaccines for UTIs that have entered the market [131]: Uro-Vaxom, an oral vaccine approved in Germany and Switzerland for the prevention of recurrent cystitis [64]; Urovac, approved across Europe for the prevention of UTIs [57]; Uromune, a sublingual immunogenic vaccine that has demonstrated significant efficacy and good safety in preventing UTIs in multiple clinical trials [68,132,133]. Among these, Uro-Vaxom (OM-89) has shown particular promise in clinical applications. This oral vaccine consists of lyophilized bacterial lysates from 18 different *E. coli* strains, the most common uropathogen. Clinical studies have demonstrated that OM-89 can significantly reduce the recurrence rate of UTIs by stimulating both systemic and mucosal immune responses [64,65,66]. Solco-Urovac, another whole-cell vaccine, employs a different administration route (intramuscular) and contains inactivated bacteria from ten uropathogenic strains (six UPEC strains, one strain of each *Proteus mirabilis*, *Klebsiella pneumoniae*, *Morganella morganii* and *Enterococcus faecalis*). Its efficacy has been demonstrated in multiple randomized controlled trials, showing a significant reduction in UTI recurrence rates compared to placebo groups [60,61]. Uromune represents an innovative approach among whole-cell vaccines, utilizing a sublingual administration route that takes advantage of the rich immune network in the oral mucosa. This vaccine contains inactivated whole cells from four bacterial species commonly causing UTIs. The sublingual route offers several advantages, including ease of administration, avoidance of first-pass metabolism, and induction of both systemic and mucosal immunity.

Initial live-attenuated vaccine candidates targeting UPEC included CP923, engineered through mutations in capsular and lipopolysaccharide O-antigen biosynthesis pathways. Intranasal administration of formalin-inactivated CP923 elicited robust humoral immunity in murine models, as evidenced by elevated serum antibody titers. However, this formulation failed to confer protection in sepsis challenge experiments [70,71]. Mechanistic analysis revealed that surface polysaccharides expressed by CP923 and other UPEC strains sterically shielded underlying protein epitopes, thereby diminishing antibody-mediated neutralization of non-carbohydrate antigens. A second attenuated construct, NU14 Δ*waaL*, was developed by disrupting the O-antigen ligase gene (*waaL*), impairing bacterial colonization within the urinary tract. Intravesical instillation of this strain in mice demonstrated efficacy in preventing bladder colonization and suppressing UPEC persistence across heterologous strains. Nevertheless, clinical translation was limited by two factors: rapid clearance of the vaccine strain from the bladder mucosa and absence of protective immunity in renal tissues [134].

### 3.2. Polysaccharide-Based Conjugate Vaccines

Bacterial polysaccharide conjugate vaccines are glycoprotein complexes prepared by purification of surface polysaccharides from pathogenic bacteria and their subsequent covalent binding to carrier proteins with significant immunogenicity, either chemically or by enzyme catalysis (see Table 2) [135,136]. These vaccines are designed to increase the immunogenicity of the polysaccharide antigen, thereby inducing a stronger immune response. The surface polysaccharides of pathogenic bacteria, such as capsule polysaccharides (CPS) and O-antigen polysaccharides (OPS), are crucial virulence factors with high species specificity [137,138]. Additionally, CPS/OPS possess immunogenicity, stimulating the body to produce protective antibodies and making them important targets for vaccine development. However, pure polysaccharide antigens can only induce T cell-independent (TI) responses, which are characterized by the production of low-affinity IgM antibodies, limited immunological memory, and consequently, short-lived protection [139]. Polysaccharide conjugate vaccines overcome this limitation by harnessing a T cell-dependent (TD) pathway [140,141]. The covalent linkage to a carrier protein allows the polysaccharide to be presented to T helper cells. This essential T-cell help drives the B cell response through the germinal center reaction, leading to isotype switching to high-affinity IgG, affinity maturation, and the generation of long-lived memory B cells and plasma cells. This mechanistic shift is why polysaccharide conjugate vaccines generate robust and durable immune protection and are considered one of the most successful vaccine forms for humans [142].

Currently, several candidate polysaccharide conjugate vaccines for UTIs, such as ExPEC4V, ExPEC9V, and ExPEC10V, have been designed to leverage the immunogenicity of CPS/OPS. While preclinical and early clinical data are promising, their overall efficacy and optimal formulation are still under investigation [73,80,143]. ExPEC4V is a polysaccharide conjugate vaccine formed by the chemical conjugation of four known O antigens (O1A, O2, O6A, and O25B) that cause UTIs with the carrier protein EPA. ExPEC4V targets 46% of clinical UPEC isolates and 47% of MDR isolates [144]. A Phase I study demonstrated that ExPEC4V has robust immunogenicity and a clinically acceptable safety profile in healthy women [77]. Furthermore, Phase II trials confirm that all tested doses of the ExPEC4V bioconjugate vaccine candidate are well-tolerated and elicit potent functional antibody responses against each of the four included serotypes [74]. ExPEC9V comprises nine O-antigen polysaccharides (serotypes O1, O2, O4, O6, O15, O16, O18, O25, and O75), covering 65% of clinical UPEC isolates and 37.4% of multidrug-resistant (MDR) isolates [143]. It is indicated for active immunization in adults aged ≥60 years to prevent first-episode invasive extraintestinal pathogenic *E. coli* disease (IED). The vaccine is currently under evaluation in an ongoing Phase I trial (NCT04899336), with results pending publication. ExPEC10V comprises ten O-antigen polysaccharides (serotypes O1A, O2, O4, O6A, O8, O15, O16, O18A, O25B, and O75), covering 72% of clinical UPEC isolates and 71% of multidrug-resistant (MDR) isolates [144]. In a randomized Phase I/IIa trial of adults with a history of urinary tract infections (UTIs), vaccination with ExPEC10V demonstrated functional opsonophagocytic killing (OPK) activity against all vaccine serotypes. Participants exhibited acceptable safety profiles and robust vaccine-induced functional immunogenicity [80]. Nevertheless, considerable heterogeneity exists in the results of candidate vaccine trials. Vaccination with ExPEC4V does not seem to reduce the recurrence of UTIs, and clinical trial results indicate that variations in the proportion of different serotypes in multivalent vaccines directly affect their antibody titers, suggesting room for optimization. Meanwhile, ExPEC9V and ExPEC10V, which are in the experimental stage, lack sufficient clinical data to support their safety and effectiveness.

### 3.3. Subunit Vaccines

Subunit vaccines represent a targeted strategy by focusing on key virulence factors such as adhesins, iron acquisition proteins, and toxins. This approach aims to elicit precise immune responses with reduced reactogenicity while maintaining strong immunogenic potential.

Adhesin-based vaccines constitute a primary focus, with the FimH-based candidate being the most advanced [145]. It utilizes truncated FimH or FimC-FimH complexes to induce IgG responses, thereby reducing UPEC colonization in the bladder mucosa. Although Phase II clinical trials were completed, its development was discontinued due to limited efficacy, which was likely attributable to FimH expression heterogeneity and insufficient antibody targeting of the critical mannose-binding domain [104,112,146]. Beyond FimH, other adhesin targets are also under exploration. These candidates, including TLR ligand-adjuvanted vaccines and PapG pili-based subunit vaccines, aim to broaden protection against various UPEC strains but remain in preclinical development (see Table 3).

Another promising avenue involves iron-scavenger-receptor-based vaccines [147,148,149]. These candidates target critical iron uptake receptors essential for bacterial survival in the host. To overcome their inherently low immunogenicity, they are often formulated with adjuvants or advanced delivery systems, such as cationized BSA or Salmonella vectors [150].

Furthermore, significant efforts have been dedicated to toxin-targeted vaccines, concentrating on HlyA and CNF1 [127]. HlyA assembles into Ca^2+^-dependent heptameric channels, inducing osmotic lysis and NLRP3 inflammasome activation, which exacerbates IL-1β/IL-18-driven inflammatory damage. Its dual cytotoxic and pro-inflammatory effects make it a high-priority target. CNF1, on the other hand, deamidates Rho GTPases, thereby disrupting cytoskeletal integrity and epithelial barrier function. It also suppresses phagocytosis by downregulating macrophage CD36 expression and promotes neutrophil-mediated tissue damage. A comprehensive summary of current vaccine candidates and their developmental status is provided in Table 3.

## 4. Manufacturing Technologies for Polysaccharide Conjugate Vaccines of UTI

Methods of polysaccharide conjugate vaccine preparation and the development of polysaccharide conjugate vaccines are pivotal for combating UTIs caused by UPEC, as LPS and CPS are key virulence factors in UPEC pathogenesis. These surface polysaccharides, particularly the O-antigen of LPS, are primary targets for vaccine design due to their immunogenicity and role in bacterial adhesion and immune evasion. This section explores traditional and biotechnological approaches to polysaccharide conjugate vaccine development, with a focus on their direct applications and potential optimizations for UTI vaccines.

### 4.1. Traditional Chemical-Based Vaccinology

The conventional chemical conjugation method has been instrumental in developing vaccines against bacterial pathogens, including UPEC. The world’s first successfully launched glycoprotein conjugate vaccine was the *Haemophilus influenzae* type b (Hib) polysaccharide-protein conjugate vaccine, which combines Hib polyribosylribitol phosphate (PRP) with tetanus toxoid (Hib-tt) and has been widely used globally to effectively prevent Hib-related diseases [151]. Since then, significant progress has been made in the development of polysaccharide-protein conjugate vaccines targeting different pathogens. Since 2000, three pneumococcal conjugate vaccines—Prevnar7^®^, Synflorix™, and Prevnar 13^®^—have been commercially licensed and put into use [152,153]. Among them, Prevnar 13^®^ is renowned for its broad protective scope, consisting of 13 different polysaccharide-protein conjugates and each serotype’s polysaccharide covalently linked to the genetically inactivated diphtheria toxoid CRM197 [152]. This strategy is now being adapted for UPEC-specific vaccines like ExPEC4V. These candidates utilize O-antigens chemically linked to carrier proteins (e.g., EPA) to enhance immunogenicity. However, this preparation process also has significant limitations. On the one hand, multi-step purification not only increases production costs but also pushes up vaccine prices. On the other hand, the randomness of chemical activation sites makes it difficult to precisely control the connection sites between polysaccharides and proteins and the number of polysaccharides attached to each protein, thereby affecting the homogeneity and batch-to-batch stability of the vaccine product. Chemical conjugation of O1A/O2/O6A/O25B polysaccharides to EPA carriers in UTI vaccines such as ExPEC4V presents challenges in batch homogeneity [154]. Typically, only the polysaccharide-to-protein content ratio can be used as the core indicator for quality control. Additionally, traditional preparation processes involve large-scale cultivation of pathogenic bacteria and extensive use of chemical reagents, which not only pose strict requirements for biosafety but also pose challenges to environmental protection, further pushing up production costs [155].

### 4.2. Biotechnology-Enabled Vaccine Manufacturing

#### 4.2.1. Oligosaccharyltransferase (OST) System

In recent years, the application of biomanufacturing methodologies in the production of polysaccharide-based conjugate vaccines has garnered significant attention [156]. The oligosaccharyltransferase (OST) system is a crucial enzymatic machinery responsible for *N*-linked and *O*-linked protein glycosylation in eukaryotic cells and some bacteria [157,158]. The glycosyltransferase PglB from *Campylobacter jejuni* is the first discovered and most widely utilized OST in glycoconjugate vaccine development, capable of catalyzing *N*-linked glycosylation reactions [159]. PglB exhibits distinctive molecular recognition properties, specifically targeting the D/E-X-N-X-S/T (X ≠ Pro) consensus sequence while employing undecaprenyl pyrophosphate (Und-PP) as its glycan carrier to mediate *N*-linked glycosylation [160]. PglL is an O-oligosaccharyltransferase derived from *Neisseria meningitidis* [161]. While belonging to the same bacterial glycosylation system as PglB, it exhibits distinct catalytic properties [162]. This enzyme specifically recognizes disordered regions on protein surfaces and transfers oligosaccharide chains linked to undecaprenyl pyrophosphate (Und-PP) to serine/threonine residues, thereby achieving O-glycosylation modifications. Unlike PglB, which strictly depends on conserved sequences, PglL demonstrates broad substrate selectivity, a characteristic that enables its crucial role in glycosylating bacterial virulence factors (such as pilin proteins). Notably, PglL and PglB together form a comprehensive bacterial glycosylation platform. This technology has been harnessed to produce homogeneous glycoconjugates for UPEC vaccines. For example, the O4 glycoprotein, synthesized using an engineered *E. coli* chassis with optimized OST activity, targets UPEC serotype O4 and has shown promise in preclinical studies [163]. Such advancements highlight the system’s utility in generating UTI-specific conjugates with improved consistency and efficacy.

#### 4.2.2. Protein-Glycan Conjugation Technique (PGCT) In Vivo

This technology achieves one-step covalent coupling of polysaccharides to target proteins within non-pathogenic *E. coli* strains. Specifically, by co-expressing the polysaccharide synthesis gene cluster, specific OST, and a carrier protein fused with the corresponding recognition sequence (sequon) in engineered bacterial strains (such as *E. coli*), covalent linkage between polysaccharides and proteins can be efficiently achieved, thereby completing the biosynthesis of polysaccharide conjugate vaccines [164]. In PGCT, the co-expression of the polysaccharide synthesis gene cluster, OST, and recognition sequence carrier protein is crucial for the covalent linkage between polysaccharides and proteins. This process leverages the bacterial glycosyltransferase system to add sugar chains in situ on recombinant proteins, generating immunogenic glycoconjugates. By coupling polysaccharide antigens from pathogen surfaces to carrier proteins, the immunogenicity and protective efficacy of vaccines can be significantly enhanced, providing a novel strategy for vaccine development. In 2005, the Feldman team successfully synthesized polysaccharide conjugate vaccines using the bacterial OST system, marking a significant milestone in the application of PGCT in vaccine research and development [165]. Compared to traditional in vitro chemical coupling methods, PGCT offers advantages such as high efficiency, strong specificity, and ease of operation. It achieves one-step coupling of polysaccharides and proteins within bacterial cells, avoiding cumbersome in vitro steps and potential toxicity issues [166,167]. Furthermore, this technology leverages the bacterial metabolism and regulatory mechanisms to optimize the expression and modification of polysaccharides and proteins, thereby improving vaccine yield and purity, and further optimizing vaccine immune responses and protective efficacy. Currently, PGCT has been widely applied in the production of UTI vaccine candidates [168].

#### 4.2.3. Metabolic Engineering-Directed Biosynthesis of Polysaccharide-Based Conjugate Vaccines

In glycoconjugate vaccines, the antibody response against pathogenic bacterial polysaccharides constitutes the fundamental mechanism of vaccine-mediated immune protection. Therefore, increasing the polysaccharide content in polysaccharide-based conjugate vaccines is critical for enhancing UTI vaccine potency. In recent years, the integration of metabolic engineering and synthetic biology technologies has provided revolutionary solutions for the efficient production of glycoconjugate vaccines [169]. Through systematic regulation of microbial metabolic networks, researchers have achieved precise directional optimization of sugar precursor synthesis [170,171]. Specifically, the rational modification of chassis strains using gene-editing tools such as CRISPR-Cas9—including knockout of competing pathway genes (e.g., *zwf*, *pgi*) to enhance pentose phosphate flux and overexpression of key sugar nucleotide synthetases (e.g., *glmU*, *galE*)—has significantly increased the production of essential sugar precursors such as UDP-GlcNAc. In terms of expression system optimization, promoter engineering and codon optimization strategies have dynamically balanced the expression of glycosyltransferases and carrier proteins, thereby improving the glycosylation efficiency of recombinant proteins. Coupled with orthogonal glycosylation systems, this strategy has enabled the biosynthesis of UPEC-specific glycoconjugates (e.g., O21-OPS glycoprotein), demonstrating its direct applicability to UTI vaccine development [172]. Currently, the combination of AI-driven metabolic modeling and automated fermentation platforms is driving this technology toward intelligent and continuous production, with the potential to achieve customized glycoconjugate vaccine manufacturing within the next five years to meet the demands of personalized immunotherapy.

### 4.3. Lessons from Other Pathogens: The Real-World Impact of Polysaccharide Conjugate Vaccines

Beyond their potential in UPEC vaccine development, polysaccharide-based conjugate vaccines have achieved landmark success in combating other encapsulated bacteria, offering both instructive parallels and critical cautions for UTI vaccine design. Notable examples include vaccines against *Haemophilus influenzae* type b (Hib) and *Streptococcus pneumoniae*. The introduction of Hib conjugate vaccines has reduced the global incidence of invasive Hib diseases, such as meningitis, by over 99%, nearly eliminating this pathogen as a public health threat [173]. Similarly, widespread administration of pneumococcal conjugate vaccines (PCVs) has dramatically decreased the incidence of pediatric pneumonia, meningitis, and bacteremia caused by vaccine-targeted serotypes [174]. However, these successes have also revealed a major challenge: serotype replacement. Following the effective use of 7-valent and 13-valent PCVs, non-vaccine serotypes of S. pneumoniae have filled the ecological niche, emerging as new dominant pathogens [175]. More concerningly, these replacement serotypes are often associated with higher levels of antibiotic resistance, thereby shifting the epidemiology of resistant infections and posing additional challenges to public health [176].

These historical precedents carry direct implications for UPEC O-antigen-targeted vaccines. With over 180 distinct O-serotypes—far exceeding the diversity of pneumococcal serotypes—UPEC presents a substantial risk of serotype replacement should a monovalent or limited-valency vaccine be deployed. Such an approach could select for infections caused by non-targeted O-serotypes, potentially compounding the problem with increased antimicrobial resistance. To preemptively address this challenge, future UPEC vaccine strategies should focus on defining and covering predominant high-risk and resistant serotypes, while also exploring highly conserved protein antigens (e.g., FimH, HlyA) to broaden protection. An integrated approach combining conserved protein targets with core O-antigen elements may offer a promising path forward, potentially overcoming serotypic limitations while minimizing the risk of driving further resistance.

The translation of such sophisticated vaccine strategies into clinical practice further necessitates the identification of specific target populations to ensure cost-effectiveness and maximize public health impact. Given the substantial cost of vaccine development and implementation, a universal vaccination strategy is neither practical nor cost-effective. Instead, a targeted approach is warranted. Primary candidates for vaccination would be well-defined high-risk populations. These include: (1) postmenopausal women with a history of recurrent UTIs; (2) individuals with spinal cord injuries or neurogenic bladder requiring intermittent catheterization; (3) patients undergoing elective urologic surgery or renal transplantation; and (4) elderly residents in long-term care facilities. The optimal timing of administration would be prophylactic, for instance, prior to the onset of risk (e.g., in younger women showing a pattern of recurrence) or scheduled before a predictable risk event, such as surgery.

### 4.4. Integrated Strategies and Future Directions for UTI Management

Prophylactic vaccination represents an effective strategy for preventing urinary tract infections (UTIs), though realizing its full protective and therapeutic potential often requires synergistic integration with alternative treatment modalities. Studies demonstrate that combining vaccines with non-antibiotic agents can yield synergistic effects, enhance protection while mitigate the risk of antimicrobial resistance [177]. For instance, co-administration of a vaccine targeting UPEC adhesins such as FimH with mannosides—small-molecule inhibitors that block FimH-mediated binding—reduces the genetic diversity of gut-colonizing UPEC strains, treats UTIs, and preserves commensal gut microbiota structure. Mannosides selectively inhibit intestinal UPEC colonization, significantly decreasing UTI incidence and recurrence [178]. Similarly, combining vaccines with probiotics such as Lactobacillus strains helps restore a healthy urogenital microbiota, thereby generating an environment less conducive to pathogen colonization [179]. Furthermore, combining vaccines with bacteriophage therapy offers a promising direction for addressing established or breakthrough infections, particularly those caused by multidrug-resistant strains, serving as a targeted rescue intervention [180].

Beyond these approaches, other non-antibiotic therapies show potential [181]. Cranberry preparations, which contain proanthocyanidins and fructose that disrupt bacterial adhesion, exhibit debated clinical efficacy due to formulation variability and inconsistent evidence for preventing catheter-associated UTIs (CAUTIs) [182]. Methenamine, which decomposes into formaldehyde in acidic urine, exerts bactericidal effects and can delay bacteriuria onset and reduce CAUTI risk, though it is contraindicated in patients with renal or hepatic impairment [183,184]. D-mannose competitively inhibits FimH-mediated bacterial adhesion; while its mechanism is well-established, clinical evidence supporting its use for CAUTI prevention remains limited [185]. Probiotics such as Lactobacillus species demonstrate preventive potential in non-catheterized populations, but their efficacy in catheterized patients remains unclear, and their ability to transiently modulate catheter biofilm communities warrants further investigation [186]. Collectively, these integrated strategies highlight that combining vaccination with non-antibiotic therapies may enhance protective outcomes while reducing the selective pressure for antimicrobial resistance.

## 5. Conclusions

To date, no vaccine for UTI prevention has been approved in the United States, but several UPEC vaccine candidates have entered clinical trial phases [76,79,80,187]. Research on UPEC vaccines primarily focuses on specific antigens and virulence factors [146]. Among them, bacterial surface polysaccharides such as CPS and OPS, serving as key virulence factors, exhibit high species specificity and immunogenicity, are capable of inducing the production of protective antibodies, thus becoming important targets for vaccine development [77]. However, pure polysaccharide antigens can only induce T cell-independent (TI) antigens and produce IgM antibodies with low affinity, failing to elicit long-lasting immune protection. Polysaccharide conjugate vaccines, formed by covalently coupling bacterial polysaccharides with immunogenic carrier proteins, can elicit T cell-dependent responses, generating high-affinity polysaccharide-specific IgG antibodies and long-lasting immune memory [140]. Notably, while polysaccharide-based conjugate vaccines rank among the most successful medical interventions historically, their translation to the UTI field remains fraught with unresolved challenges, including the extensive serotype diversity of UPEC and the potential for serotype replacement following vaccination.

While the current review focuses on UPEC as the primary pathogen, the glycoconjugate platform is inherently versatile and amenable to the development of multivalent vaccines. A strategic approach for a broadly protective UTI vaccine could involve creating a multivalent formulation that targets the O-antigens of the most prevalent uropathogens, such as *Klebsiella pneumoniae*, *Proteus mirabilis*, and *Enterococcus faecalis*, in addition to dominant UPEC serotypes. This would transform the vaccine from a pathogen-specific intervention into a more useful, multipurpose prophylactic tool against the broader spectrum of pathogens responsible for both community-acquired and healthcare-associated UTIs.

With advancements in biotechnology, in vivo protein-glycan coupling technology has emerged, utilizing biosynthetic mechanisms to achieve efficient and precise conjugation of polysaccharides and proteins, providing new avenues for the synthesis and optimization of UTI vaccines [168]. The future optimization directions can be summarized as follows: (1) Directed evolution of enzymes. With the discovery of various glycosyltransferases with broad substrate specificity, the targets for the biological preparation of polysaccharide conjugate vaccines have expanded from OPS of Gram-negative bacteria to CPS of some Gram-positive bacteria and special polysaccharides. With further research, full coverage of extracellular polysaccharides of pathogenic bacteria is expected to be achieved. Meanwhile, utilizing structural biology techniques to continuously analyze the structures and functions of several different glycosyltransferases, it is hoped that through enzyme engineering, the functions of different glycosyltransferases can be integrated, enabling a single enzyme to recognize multiple target polysaccharides with different characteristics. (2) Design of carrier proteins. Currently, the carrier proteins primarily used in vaccine development still focus on Tetanus Toxoid (TT) and Diphtheria Toxoid (DT) [188]. However, when the same carrier protein is used for diverse polysaccharide-based conjugate vaccines, it may trigger mutual interference in immune responses, thereby weakening the efficacy of subsequent vaccinations [189,190]. To this end, novel carrier proteins such as CRM197, bacterial outer membrane proteins (e.g., MBP, Haemophilus influenzae D protein), and Pseudomonas aeruginosa exotoxin A (EPA) have been developed to enrich carrier diversity [191,192]. In recent years, nanoscale particulate vaccines have received widespread attention due to their significant advantages in antigen delivery efficiency and immunostimulatory capability [193]. Pan et al. innovatively fused a trimer domain to the terminus of the Cholera Toxin B Subunit (CTB), successfully constructing sixty-mer protein particles with a particle size of approximately 20–30 nm [169]. Polysaccharide conjugate vaccines prepared using this as a carrier exhibited superiority compared to recombinant EPA (rEPA) carrier vaccines, enhancing the immune efficacy of polysaccharide conjugate vaccines [162]. However, the long-term safety and manufacturability of these complex nanostructures must be thoroughly evaluated before clinical translation. In addition, the “two-component” immunization strategy integrates glycan and protein components from the same pathogen, wherein the carrier protein not only serves as a transport vehicle but also exerts a more active immunomodulatory role, opening new avenues for vaccine design and development. (3) Modification of chassis cells. Many scholars have developed glycoengineering platforms for sugar conjugate vaccines using *E. coli* as the host. Through these platforms, bacterial sugar conjugate vaccines can be rapidly prepared. For example, Pan et al.’s Nano-B5 platform utilizes the self-assembly capabilities of bacterial AB5 toxins and the pentamer domain of non-natural trimer peptides to produce various nano-vaccines in vivo. Wang et al. constructed a specific *E. coli* chassis MG1655 for O-glycosylation, successfully synthesizing O21 glycoprotein targeting the UPEC O21 serotype by optimizing metabolic pathways and enhancing UDP-sugar precursor synthesis [163,172]. This not only provides new candidate molecules for UTI vaccine development but also demonstrates the enormous potential of sugar platforms and optimization strategies based on bacterial cells in the field of biosynthesis. These technological advancements provide the essential foundation for developing the multivalent vaccine strategy discussed above.

## Figures and Tables

**Table 1:** Types and characteristics of whole-cell vaccines for UTI-preventive UPEC vaccination.

Vaccine Type	Name	Development Stage	Constituents	Routes of Administration	Protection Effects	Side Effects
Inactivated vaccines	Solco-Urovac^®^ [57,58,59]	Marketed	10 strains of killed uropathogens	Vaginal inoculation	Reduced recurrent UTIs in women	Headache, gastrointestinal disturbances, and vaginitis
	StroVac^®^ [60,61,62,63]	Marketed	10 strains in different configuration.	Subcutaneous	Significantly reduce the number of clinically relevant UTIs	Redness and systemic reactions (such as fatigue, etc.)
	Uro-Vaxom (OM89^®^) [64,65,66,67]	Marketed	Membrane proteins from 18 strains of UPEC	Oral capsule	Effectively prevents recurrent UTIs in women	Requires continuous use for three months
	Urvakol & Urostim [68,69]	Preclinical /Clinical	Inactivated uropathogens	Oral tablet	high stimulation of systemic and mucosal immune responses	Protective efficacy not proven
Attenuated vaccines	CP923 [70,71]	Experimental/Preclinical	Mutations in capsule and O antigen from LPS in UPEC	Intranasal inoculation	Produced significant humoral immune responses in serum	Lack of protection in sepsis model
	NU14 ΔwaaL [72]	Experimental/Preclinical	Deletion in gene encoding O antigen ligase	Inoculation into the bladder of mice	Protected the bladder	Lack of kidney protection

**Table 2:** Types and Characteristics of Polysaccharide-based Conjugate Vaccines for UTI-Preventive UPEC Vaccination.

Name	Development Stage	Constituents	Routes of Administration	Protection Effects	Side Effects
ExPEC4V (NCT03500679) [73,74,75,76,77]	Preclinical	O-antigen polysaccharides of ExPEC serotypes O1A, O2, O6A, and O25B	Subcutaneous	Demonstrated a favorable safety profile in clinical trials, with no serious or adverse events associated with the vaccine	Failed to reach statistical significance in human clinical trials
ExPEC9V (NCT04899336) [78,79]	clinical trial stage	O-antigen polysaccharides of ExPEC serotypes O1, O2, O4, O6, O15, O16, O18, O25 and O75	Subcutaneous	Prevention of Invasive ExPEC Disease in Adults Aged 60 Years and Older with a History of UTI in the Past 2 Years	Trial ongoing (recruiting), expected to end in 2029
ExPEC10V (NCT03819049) [80]	Phase 1/2a Clinical Trial	O-antigen polysaccharides of ExPEC serotypes O1A, O2, O4, O6A, O8, O15, O16, O18A, O25B, and O75	Subcutaneous	Elicited a robust immunogenic IgG antibody response across all tested vaccine serotypes, demonstrated functional opsonophagocytic killing of *E. coli* strains for 9 of the 10 vaccine serotypes	Solicited systemic adverse events (AEs) such as myalgia, headache, fever, etc., were reported; unsolicited AEs and serious AEs (SAEs) were also observed

**Table 3:** Types and Characteristics of subunit vaccines for UTI-Preventive UPEC Vaccination.

Vaccine Type	Name	Development Stage	Constituents	Routes	Protection Effects	Side Effects
Fimbrial adhesin vaccines	FimH-based Vaccine [112]	Phase II clinical trial	Truncated FimH, FimC-FimH Complex	Subcutaneous	Reduced colonization of UPEC strains in bladder; Induced IgG response	Ineffectiveness; Variations in FimH expression; Antibodies do not target mannose-binding region
	Fusion FimH.FliC with CT Adjuvant [113]	Preclinical	Fusion of FimH adhesin with flagellin (FliC), with CT adjuvant	Intranasal	Induced humoral and mixed Th1/Th2 responses; Significant protection against experimental infection	Not specified
	DNA Vaccine [114,115]	Preclinical	Prokaryotic plasmid vector encoding FimH antigen	Subcutaneous	Increased cell proliferation and production of IFN-γ cytokine; Reduced bacterial load in a bladder challenge model	Not specified
	Polyvalent Vaccine (FimH and MrpH) [116,117]	Preclinical	Fusion of FimH and MrpH adhesins of UPEC and Proteus mirabilis	Subcutaneous	Induced humoral and cellular responses; Reduced bacterial load in the bladder and kidneys	Not specified
	S Pili-based Vaccine [118]	Preclinical	S pili antigen	Subcutaneous	Protect mice against lethal sepsis in a mice model	Not specified
	PapG Pili-based Subunit Vaccine [119,120,121]	Preclinical	PapG pili antigen	Subcutaneous	Inhibited UPEC in the kidney of mice and monkey models	Lack of protection in the bladder
Non-fimbrial adhesin vaccines	TLR Ligand-Adjuvanted Vaccines [122]	Preclinical	Fusion of FimH adhesin with FliC of UPEC	Subcutaneous, Intranasal	Induced humoral and cellular responses; Maintained IgG response for 8 months	Not specified
	TosA-based Vaccine [88]	Preclinical	Mutation in the gene encoding TosA in UPEC strain	Subcutaneous	Caused a defect in the ability of UPEC colonization	Not specified
	UpaG Auto-transporter-based Vaccine [123]	Preclinical	UpaG protein	Subcutaneous	Provided protection after active and passive immunization in a sepsis model	Not specified
	FdeC Adhesin-based Vaccine [124]	Preclinical	FdeC adhesin	Intranasal	Protected colonization of UPEC strain in the kidneys	Not specified
Iron-scavenger-receptor-based vaccines	Various Iron Absorption Receptor-Based Vaccines [125]	Preclinical	Iron absorption receptors in conjunction with adjuvants or delivery systems	Subcutaneous, Intranasal	Reduced bacterial colonization in the bladder, kidneys, spleen.	Low immunogenicity of subunit recombinant vaccines; need for further clinical trials to assess safety and efficacy
Toxins-based vaccines	HlyA-based vaccine [126]	Preclinical	Purified hemolysin (HlyA) from UPEC supernatant	Subcutaneous	Resulted in a nonsignificant decrease in kidney damage in mice	Not specified
	Recombinant HlyA vaccine (ecp_3827 candidate) [127]	Preclinical	Recombinant hemolysin (ecp_3827 candidate) amplified from UPEC strain 536	Subcutaneous	Provided 76% protection in a sepsis mice model	Not specified
	Mutation-based vaccine (CNF1 and HlyA) [128,129,130]	Preclinical	Mutations in CNF1 and HlyA toxin genes in UPEC strain	Subcutaneous	Reduced cystitis in mice compared to control; CNF1mutation reduced prostatitis severity	Not specified
	Toxoid-based vaccine (HlyA and CNF1) [128,129]	Preclinical	Toxoids of HlyA and CNF1	Subcutaneous	Significant antibody response and reduced bacterial load in urine and bladder; CNF1-vaccinated mice showed increased antibody titer	Inefficiency of CNF1 vaccination due to inadequate titer of neutralizing antibodies in the bladder

## Data Availability

No new data were created or analyzed in this study. Data sharing is not applicable to this article.

## References

[1] Hof H. (2017). Candiduria! What now? Therapy of urinary tract infections with Candida. Der Urol..

[2] Hannan T.J., Totsika M., Mansfield K.J., Moore K.H., Schembri M.A., Hultgren S.J. (2012). Host-pathogen checkpoints and population bottlenecks in persistent and intracellular uropathogenic *Escherichia coli* bladder infection. FEMS Microbiol. Rev..

[3] Flores-Mireles A.L., Walker J.N., Caparon M., Hultgren S.J. (2015). Urinary tract infections: Epidemiology, mechanisms of infection and treatment options. Nat. Rev. Microbiol..

[4] Hanna-Wakim R.H., Ghanem S.T., El Helou M.W., Khafaja S.A., Shaker R.A., Hassan S.A., Saad R.K., Hedari C.P., Khinkarly R.W., Hajar F.M. (2015). Epidemiology and characteristics of urinary tract infections in children and adolescents. Front. Cell. Infect. Microbiol..

[5] Zorc J.J., Levine D.A., Platt S.L., Dayan P.S., Macias C.G., Krief W., Schor J., Bank D., Shaw K.N., Kuppermann N. (2005). Clinical and demographic factors associated with urinary tract infection in young febrile infants. Pediatrics.

[6] Shaikh N., Morone N.E., Bost J.E., Farrell M.H. (2008). Prevalence of urinary tract infection in childhood: A meta-analysis. Pediatr. Infect. Dis. J..

[7] Kanellopoulos T.A., Salakos C., Spiliopoulou I., Ellina A., Nikolakopoulou N.M., Papanastasiou D.A. (2006). First urinary tract infection in neonates, infants and young children: A comparative study. Pediatr. Nephrol..

[8] Mattoo T.K., Shaikh N., Nelson C.P. (2021). Contemporary Management of Urinary Tract Infection in Children. Pediatrics.

[9] Tullus K., Shaikh N. (2020). Urinary tract infections in children. Lancet.

[10] Hellström A., Hanson E., Hansson S., Hjälmås K., Jodal U. (1991). Association between urinary symptoms at 7 years old and previous urinary tract infection. Arch. Dis. Child..

[11] Foxman B. (2014). Urinary tract infection syndromes: Occurrence, recurrence, bacteriology, risk factors, and disease burden. Infect. Dis. Clin. N. Am..

[12] Kaur R., Kaur R. (2021). Symptoms, risk factors, diagnosis and treatment of urinary tract infections. Postgrad. Med. J..

[13] Hooton T.M. (2012). Clinical practice. Uncomplicated urinary tract infection. N. Engl. J. Med..

[14] Schappert S.M., Rechtsteiner E.A. (2011). Ambulatory medical care utilization estimates for 2007. Vital Health Stat. 13.

[15] Chen Y.-C., Lee W.-C., Chuang Y.-C. (2023). Emerging non-antibiotic options targeting uropathogenic mechanisms for recurrent uncomplicated urinary tract infection. Int. J. Mol. Sci..

[16] Worby C.J., Schreiber IV H.L., Straub T.J., van Dijk L.R., Bronson R.A., Olson B.S., Pinkner J.S., Obernuefemann C.L., Muñoz V.L., Paharik A.E. (2022). Longitudinal multi-omics analyses link gut microbiome dysbiosis with recurrent urinary tract infections in women. Nat. Microbiol..

[17] Suskind A.M., Saigal C.S., Hanley J.M., Lai J., Setodji C.M., Clemens J.Q. (2016). Incidence and Management of Uncomplicated Recurrent Urinary Tract Infections in a National Sample of Women in the United States. Urology.

[18] O’Brien V.P., Hannan T.J., Nielsen H.V., Hultgren S.J. (2016). Drug and Vaccine Development for the Treatment and Prevention of Urinary Tract Infections. Microbiol. Spectr..

[19] Alam M., Anwar M., Akhtar M., Alam P., Mohammad A., Almutairy A., Nazmi A., Mukherjee T. (2024). A systematic review of recent advances in urinary tract infection interventions and treatment technology. Eur. Rev. Med. Pharmacol. Sci..

[20] Stamm W.E., Hooton T.M., Johnson J.R., Johnson C., Stapleton A., Roberts P.L., Moseley S.L., Fihn S.D. (1989). Urinary tract infections: From pathogenesis to treatment. J. Infect. Dis..

[21] Johansen T.E., Botto H., Cek M., Grabe M., Tenke P., Wagenlehner F.M., Naber K.G. (2011). Critical review of current definitions of urinary tract infections and proposal of an EAU/ESIU classification system. Int. J. Antimicrob. Agents.

[22] Foxman B. (2002). Epidemiology of urinary tract infections: Incidence, morbidity, and economic costs. Am. J. Med..

[23] Park J., Torosis M., Kim J.H., Ackerman A.L. (2024). U.S. primary care physician perceptions on barriers to providing guideline-driven care for UTI and recurrent UTI: A qualitative study. BMC Prim. Care.

[24] Reissier S., Penven M., Amara M., Dortet L., Riverain E., Caspar Y., Degand N., Farfour E., Corvec S., Barraud O. (2025). Bacterial epidemiology and antibiotic resistance rates in male urinary tract infections in France, 2019–2023. Infect. Dis. Now.

[25] Gupta K., Grigoryan L., Trautner B. (2017). Urinary Tract Infection. Ann. Intern. Med..

[26] Chu C.M., Lowder J.L. (2018). Diagnosis and treatment of urinary tract infections across age groups. Am. J. Obstet. Gynecol..

[27] Muller A.E., Verhaegh E.M., Harbarth S., Mouton J.W., Huttner A. (2017). Nitrofurantoin’s efficacy and safety as prophylaxis for urinary tract infections: A systematic review of the literature and meta-analysis of controlled trials. Clin. Microbiol. Infect..

[28] Zykov I.N., Frimodt-Møller N., Småbrekke L., Sundsfjord A., Samuelsen Ø. (2020). Efficacy of mecillinam against clinical multidrug-resistant *Escherichia coli* in a murine urinary tract infection model. Int. J. Antimicrob. Agents.

[29] Milano A., Sulejmani A., Intra J., Sala M.R., Leoni V., Carcione D. (2022). Antimicrobial Resistance Trends of *Escherichia coli* Isolates from Outpatient and Inpatient Urinary Infections over a 20-Year Period. Microb. Drug Resist..

[30] Car J. (2006). Urinary tract infections in women: Diagnosis and management in primary care. BMJ.

[31] Chardavoyne P.C., Kasmire K.E. (2020). Appropriateness of Antibiotic Prescriptions for Urinary Tract Infections. West. J. Emerg. Med..

[32] Holm A., Cordoba G., Aabenhus R. (2019). Prescription of antibiotics for urinary tract infection in general practice in Denmark. Scand. J. Prim. Health Care.

[33] Gupta K., Hooton T.M., Naber K.G., Wullt B., Colgan R., Miller L.G., Moran G.J., Nicolle L.E., Raz R., Schaeffer A.J. (2011). International clinical practice guidelines for the treatment of acute uncomplicated cystitis and pyelonephritis in women: A 2010 update by the Infectious Diseases Society of America and the European Society for Microbiology and Infectious Diseases. Clin. Infect. Dis..

[34] Anger J., Lee U., Ackerman A.L., Chou R., Chughtai B., Clemens J.Q., Hickling D., Kapoor A., Kenton K.S., Kaufman M.R. (2019). Recurrent Uncomplicated Urinary Tract Infections in Women: AUA/CUA/SUFU Guideline. J. Urol..

[35] Kodner C.M., Thomas Gupton E.K. (2010). Recurrent urinary tract infections in women: Diagnosis and management. Am. Acad. Fam. Physicians.

[36] Brubaker L., Carberry C., Nardos R., Carter-Brooks C., Lowder J.L. (2018). American Urogynecologic Society Best-Practice Statement: Recurrent Urinary Tract Infection in Adult Women. Female Pelvic Med. Reconstr. Surg..

[37] Tucker A. (2023). P16 UTI Friday: A review of antibiotic prophylaxis in the management of recurrent UTI in primary care. JAC-Antimicrob. Resist..

[38] Alrosan S., Al Mse’adeen M., Alkhawaldeh I.M., Mishael J., Aljarab’ah N., Aljarajreh M., Yamin M., Abu-Jeyyab M. (2023). An Audit to Reevaluate the Adherence to the Guidelines in Patients with Urinary Tract Infection at the Al-Karak Hospital in Jordan. Cureus.

[39] Fisher S.J., Graham C., Kennard J., Jonker L. (2023). Management of urinary tract infections in the community: A clinical audit and patient survey. BJGP Open.

[40] Clark A.W., Durkin M.J., Olsen M.A., Keller M., Ma Y., O’Neil C.A., Butler A.M. (2021). Rural-urban differences in antibiotic prescribing for uncomplicated urinary tract infection. Infect. Control. Hosp. Epidemiol..

[41] Langner J.L., Chiang K.F., Stafford R.S. (2021). Current prescribing practices and guideline concordance for the treatment of uncomplicated urinary tract infections in women. Am. J. Obstet. Gynecol..

[42] Bedri A., Mulderij-Jansen V., Aits I., Berends M., Freitag M.H., van der Worp H., Glasner C., Blanker M.H. (2025). General practitioners’ perspectives on diagnosis and treatment of uncomplicated urinary tract infections: A qualitative study in the Northern Dutch–German cross-border region. Eur. J. Gen. Pract..

[43] Llor C., Bjerrum L. (2014). Antimicrobial resistance: Risk associated with antibiotic overuse and initiatives to reduce the problem. Ther. Adv. Drug Saf..

[44] Prestinaci F., Pezzotti P., Pantosti A. (2015). Antimicrobial resistance: A global multifaceted phenomenon. Pathog. Glob. Health.

[45] Mak Q., Greig J., Dasgupta P., Malde S., Raison N. (2024). Bacterial vaccines for the management of recurrent urinary tract infections: A systematic review and meta-analysis. Eur. Urol. Focus.

[46] Flores C., Rohn J.L. (2025). Bacterial adhesion strategies and countermeasures in urinary tract infection. Nat. Microbiol..

[47] Timm M.R., Russell S.K., Hultgren S.J. (2025). Urinary tract infections: Pathogenesis, host susceptibility and emerging therapeutics. Nat. Rev. Microbiol..

[48] Klein R.D., Hultgren S.J. (2020). Urinary tract infections: Microbial pathogenesis, host-pathogen interactions and new treatment strategies. Nat. Rev. Microbiol..

[49] Eto D.S., Jones T.A., Sundsbak J.L., Mulvey M.A. (2007). Integrin-mediated host cell invasion by type 1–piliated uropathogenic *Escherichia coli*. PLoS Pathog..

[50] Wu X.-R., Sun T.-T., Medina J.J. (1996). In vitro binding of type 1-fimbriated *Escherichia coli* to uroplakins Ia and Ib: Relation to urinary tract infections. Proc. Natl. Acad. Sci. USA.

[51] Pang Y., Cheng Z., Zhang S., Li S., Li X., Li X., Zhang X., Li X., Feng Y., Cui H. (2022). Bladder epithelial cell phosphate transporter inhibition protects mice against uropathogenic *Escherichia coli* infection. Cell Rep..

[52] Mittal S., Sharma M., Chaudhary U. (2015). Biofilm and multidrug resistance in uropathogenic *Escherichia coli*. Pathog. Glob. Health.

[53] Beebout C.J., Robertson G.L., Reinfeld B.I., Blee A.M., Morales G.H., Brannon J.R., Chazin W.J., Rathmell W.K., Rathmell J.C., Gama V. (2022). Uropathogenic *Escherichia coli* subverts mitochondrial metabolism to enable intracellular bacterial pathogenesis in urinary tract infection. Nat. Microbiol..

[54] Tamadonfar K.O., Omattage N.S., Spaulding C.N., Hultgren S.J. (2019). Reaching the end of the line: Urinary tract infections. Microbiol. Spectr..

[55] Medina M., Castillo-Pino E. (2019). An introduction to the epidemiology and burden of urinary tract infections. Ther. Adv. Urol..

[56] Abe C.M., Salvador F.A., Falsetti I.N., Vieira M.A., Blanco J., Blanco J.E., Blanco M., Machado A.M., Elias W.P., Hernandes R.T. (2008). Uropathogenic *Escherichia coli* (UPEC) strains may carry virulence properties of diarrhoeagenic *E. coli*. FEMS Immunol. Med. Microbiol..

[57] Uehling D.T., Hopkins W.J., Elkahwaji J.E., Schmidt D.M., Leverson G.E. (2003). Phase 2 clinical trial of a vaginal mucosal vaccine for urinary tract infections. J. Urol..

[58] Kochiashvili D., Khuskivadze A., Kochiashvili G., Koberidze G., Kvakhajelidze V. (2014). Role of the bacterial vaccine Solco-Urovac^®^ in treatment and prevention of recurrent urinary tract infections of bacterial origin. Georgian Med. News.

[59] Uehling D.T., Hopkins W.J., Dahmer L.A., Balish E. (1994). Phase I clinical trial of vaginal mucosal immunization for recurrent urinary tract infection. J. Urol..

[60] Nestler S. (2024). Harnwegsinfektprophylaxe mit StroVac. Uro-News.

[61] Nestler S., Grüne B., Schilchegger L., Suna A., Perez A., Neisius A. (2021). Efficacy of vaccination with StroVac for recurrent urinary tract infections in women: A comparative single-centre study. Int. Urol. Nephrol..

[62] Nestler S., Peschel C., Horstmann A.H., Vahlensieck W., Fabry W., Neisius A. (2023). Correction to: Prospective multicentre randomized double-blind placebo-controlled parallel group study on the efficacy and tolerability of StroVac^®^ in patients with recurrent symptomatic uncomplicated bacterial urinary tract infections. Int. Urol. Nephrol..

[63] Nestler S., Grüne B., Schilchegger L., Neisius A. (2021). P0176—Prophylaxis for recurrent urinary tract infections in women with StroVac—Efficacy and patient’s compliance in a 2-year follow-up. Eur. Urol..

[64] Wade D., Cooper J., Derry F., Taylor J. (2019). Uro-Vaxom^®^ versus placebo for the prevention of recurrent symptomatic urinary tract infections in participants with chronic neurogenic bladder dysfunction: A randomised controlled feasibility study. Trials.

[65] Cruz F., Dambros M., Naber K.G., Bauer H.W., Cozma G. (2009). Recurrent Urinary Tract Infections: Uro-Vaxom^®^, a New Alternative. Eur. Urol. Suppl..

[66] Porto B.C., Almeida A.S., Terada B.D., Gonçalves F.G., Passerotti C.C., Sardenberg R.A., Otoch J.P., Cruz J.A.D. (2025). Uro-vaxom (OM-89) for chronic UTI prevention: An updated meta-analysis, meta-regression and trial sequential analysis of recent clinical evidence. Minerva Urol. Nephrol..

[67] Magasi P., Pánovics J., Illés A., Nagy M. (1994). Uro-Vaxom and the management of recurrent urinary tract infection in adults: A randomized multicenter double-blind trial. Eur. Urol..

[68] Magistro G., Stief C.G. (2019). Vaccine Development for Urinary Tract Infections: Where Do We Stand?. Eur. Urol. Focus.

[69] Marinova S., Nenkov P., Markova R., Nikolaeva S., Kostadinova R., Mitov I., Vretenarska M. (2005). Cellular and humoral systemic and mucosal immune responses stimulated by an oral polybacterial immunomodulator in patients with chronic urinary tract infections. Int. J. Immunopathol. Pharmacol..

[70] Russo T.A., Beanan J.M., Olson R., Genagon S.A., MacDonald U., Cope J.J., Davidson B.A., Johnston B., Johnson J.R. (2007). A killed, genetically engineered derivative of a wild-type extraintestinal pathogenic *E. coli* strain is a vaccine candidate. Vaccine.

[71] Russo T.A., Beanan J.M., Olson R., MacDonald U., Cope J.J. (2009). Capsular polysaccharide and the O-specific antigen impede antibody binding: A potential obstacle for the successful development of an extraintestinal pathogenic *Escherichia coli* vaccine. Vaccine.

[72] Billips B.K., Forrestal S.G., Rycyk M.T., Johnson J.R., Klumpp D.J., Schaeffer A.J. (2007). Modulation of host innate immune response in the bladder by uropathogenic *Escherichia coli*. Infect. Immun..

[73] Saade E., Gravenstein S., Donskey C.J., Wilson B., Spiessens B., Abbanat D., Poolman J., Ibarra de Palacios P., Hermans P. (2020). Characterization of *Escherichia coli* isolates potentially covered by ExPEC4V and ExPEC10V, that were collected from post-transrectal ultrasound-guided prostate needle biopsy invasive urinary tract and bloodstream infections. Vaccine.

[74] Frenck R.W., Ervin J., Chu L., Abbanat D., Spiessens B., Go O., Haazen W., van den Dobbelsteen G., Poolman J., Thoelen S. (2019). Safety and immunogenicity of a vaccine for extra-intestinal pathogenic *Escherichia coli* (ESTELLA): A phase 2 randomised controlled trial. Lancet Infect. Dis..

[75] Smith W.B., Abbanat D., Spiessens B., Go O., Haazen W., de Rosa T., Fae K., Poolman J., Thoelen S., de Palacios P.I. (2019). 2712. Safety and Immunogenicity of two Doses of ExPEC4V Vaccine Against Extraintestinal Pathogenic *Escherichia coli* Disease in Healthy Adult Participants. Open Forum Infect. Dis..

[76] Inoue M., Ogawa T., Tamura H., Hagiwara Y., Saito Y., Abbanat D., van den Dobbelsteen G., Hermans P., Thoelen S., Poolman J. (2018). Safety, tolerability and immunogenicity of the ExPEC4V (JNJ-63871860) vaccine for prevention of invasive extraintestinal pathogenic *Escherichia coli* disease: A phase 1, randomized, double-blind, placebo-controlled study in healthy Japanese participants. Hum. Vaccines Immunother..

[77] Huttner A., Gambillara V. (2018). The development and early clinical testing of the ExPEC4V conjugate vaccine against uropathogenic *Escherichia coli*. Clin. Microbiol. Infect..

[78] Del Bino L., Østerlid K.E., Wu D.-Y., Nonne F., Romano M.R., Codée J., Adamo R. (2022). Synthetic Glycans to Improve Current Glycoconjugate Vaccines and Fight Antimicrobial Resistance. Chem. Rev..

[79] Janssen Research & Development, LLC (2021). Clinical Trial: A Study of Vaccination with 9-valent Extraintestinal Pathogenic *Escherichia coli* Vaccine (ExPEC9V) in the Prevention of Invasive Extraintestinal Pathogenic *Escherichia coli* Disease in Adults Aged 60 Years and Older with a History of Urinary Tract Infection in the Past 2 Years. US Fed News Service, Including US State News.

[80] Fierro C.A., Sarnecki M., Spiessens B., Go O., Day T.A., Davies T.A., van den Dobbelsteen G., Poolman J., Abbanat D., Haazen W. (2024). A randomized phase 1/2a trial of ExPEC10V vaccine in adults with a history of UTI. npj Vaccines.

[81] Behzadi P. (2020). Classical chaperone-usher (CU) adhesive fimbriome: Uropathogenic *Escherichia coli* (UPEC) and urinary tract infections (UTIs). Folia Microbiol..

[82] Ong C.L., Beatson S.A., Totsika M., Forestier C., McEwan A.G., Schembri M.A. (2010). Molecular analysis of type 3 fimbrial genes from *Escherichia coli*, Klebsiella and Citrobacter species. BMC Microbiol..

[83] Zavialov A., Zav’yalova G., Korpela T., Zav’yalov V. (2007). FGL chaperone-assembled fimbrial polyadhesins: Anti-immune armament of Gram-negative bacterial pathogens. FEMS Microbiol. Rev..

[84] Behzadi E., Behzadi P. (2016). The role of toll-like receptors (TLRs) in urinary tract infections (UTIs). Cent. Eur. J. Urol..

[85] Spaulding C.N., Klein R.D., Ruer S., Kau A.L., Schreiber H.L., Cusumano Z.T., Dodson K.W., Pinkner J.S., Fremont D.H., Janetka J.W. (2017). Selective depletion of uropathogenic *E. coli* from the gut by a FimH antagonist. Nature.

[86] Lund B., Lindberg F., Marklund B.I., Normark S. (1987). The PapG protein is the alpha-D-galactopyranosyl-(1----4)-beta-D-galactopyranose-binding adhesin of uropathogenic *Escherichia coli*. Proc. Natl. Acad. Sci. USA.

[87] Wright K.J., Hultgren S.J. (2006). Sticky fibers and uropathogenesis: Bacterial adhesins in the urinary tract. Future Microbiol..

[88] Vigil P.D., Stapleton A.E., Johnson J.R., Hooton T.M., Hodges A.P., He Y., Mobley H.L. (2011). Presence of putative repeat-in-toxin gene *tosA* in *Escherichia coli* predicts successful colonization of the urinary tract. mBio.

[89] Engstrom M.D., Alteri C.J., Mobley H.L. (2014). A conserved PapB family member, TosR, regulates expression of the uropathogenic *Escherichia coli* RTX nonfimbrial adhesin TosA while conserved LuxR family members TosE and TosF suppress motility. Infect. Immun..

[90] Johnson J.R., Jelacic S., Schoening L.M., Clabots C., Shaikh N., Mobley H.L., Tarr P.I. (2005). The IrgA homologue adhesin Iha is an *Escherichia coli* virulence factor in murine urinary tract infection. Infect. Immun..

[91] Uhlén P., Laestadius Å., Jahnukainen T., Söderblom T., Bäckhed F., Celsi G., Brismar H., Normark S., Aperia A., Richter-Dahlfors A. (2000). α-Haemolysin of uropathogenic *E. coli* induces Ca^2+^ oscillations in renal epithelial cells. Nature.

[92] Skaar E.P. (2010). The battle for iron between bacterial pathogens and their vertebrate hosts. PLoS Pathog..

[93] Valdebenito M., Bister B., Reissbrodt R., Hantke K., Winkelmann G. (2005). The detection of salmochelin and yersiniabactin in uropathogenic *Escherichia coli* strains by a novel hydrolysis-fluorescence-detection (HFD) method. Int. J. Med. Microbiol..

[94] Reigstad C.S., Hultgren S.J., Gordon J.I. (2007). Functional genomic studies of uropathogenic *Escherichia coli* and host urothelial cells when intracellular bacterial communities are assembled. J. Biol. Chem..

[95] Schwartz L., de Dios Ruiz-Rosado J., Stonebrook E., Becknell B., Spencer J.D. (2023). Uropathogen and host responses in pyelonephritis. Nat. Rev. Nephrol..

[96] Raetz C.R.H., Whitfield C. (2002). Lipopolysaccharide Endotoxins. Annu. Rev. Biochem..

[97] Maldonado R.F., Sá-Correia I., Valvano M.A. (2016). Lipopolysaccharide modification in Gram-negative bacteria during chronic infection. FEMS Microbiol. Rev..

[98] Cohen D., Atsmon J., Artaud C., Meron-Sudai S., Gougeon M.L., Bialik A., Goren S., Asato V., Ariel-Cohen O., Reizis A. (2021). Safety and immunogenicity of a synthetic carbohydrate conjugate vaccine against Shigella flexneri 2a in healthy adult volunteers: A phase 1, dose-escalating, single-blind, randomised, placebo-controlled study. Lancet Infect. Dis..

[99] Reinhardt A., Yang Y., Claus H., Pereira C.L., Cox A.D., Vogel U., Anish C., Seeberger P.H. (2015). Antigenic potential of a highly conserved Neisseria meningitidis lipopolysaccharide inner core structure defined by chemical synthesis. Chem. Biol..

[100] Laird R.M., Ma Z., Dorabawila N., Pequegnat B., Omari E., Liu Y., Maue A.C., Poole S.T., Maciel M., Satish K. (2018). Evaluation of a conjugate vaccine platform against enterotoxigenic *Escherichia coli* (ETEC), *Campylobacter jejuni* and Shigella. Vaccine.

[101] Hug I., Feldman M.F. (2010). Analogies and homologies in lipopolysaccharide and glycoprotein biosynthesis in bacteria. Glycobiology.

[102] Micoli F., Del Bino L., Alfini R., Carboni F., Romano M.R., Adamo R. (2019). Glycoconjugate vaccines: Current approaches towards faster vaccine design. Expert Rev. Vaccines.

[103] Dow J.M., Mauri M., Scott T.A., Wren B.W. (2020). Improving protein glycan coupling technology (PGCT) for glycoconjugate vaccine production. Expert Rev. Vaccines.

[104] Hasanzadeh S., Habibi M., Shokrgozar M.A., Ahangari Cohan R., Ahmadi K., Asadi Karam M.R., Bouzari S. (2020). In silico analysis and in vivo assessment of a novel epitope-based vaccine candidate against uropathogenic *Escherichia coli*. Sci. Rep..

[105] Shah C., Baral R., Bartaula B., Shrestha L.B. (2019). Virulence factors of uropathogenic *Escherichia coli* (UPEC) and correlation with antimicrobial resistance. BMC Microbiol..

[106] Rasoulinasab M., Shahcheraghi F., Feizabadi M.M., Nikmanesh B., Hajihasani A., Sabeti S., Aslani M.M. (2021). Distribution of Pathogenicity Island Markers and H-Antigen Types of *Escherichia coli* O25b/ST131 Isolates from Patients with Urinary Tract Infection in Iran. Microb. Drug Resist..

[107] Gao M., Zhao T., Zhang C., Li P., Wang J., Han J., Zhang N., Pang B., Liu S. (2023). Ferritinophagy-mediated iron competition in RUTIs: Tug-of-war between UPEC and host. Biomed. Pharmacother..

[108] Griffith D.P., Musher D.á. (1976). Urease: Principal cause of infection stones. Urolithiasis Research.

[109] Armbruster C.E., Mobley H.L. (2012). Merging mythology and morphology: The multifaceted lifestyle of *Proteus mirabilis*. Nat. Rev. Microbiol..

[110] Stickler D. (2014). Clinical complications of urinary catheters caused by crystalline biofilms: Something needs to be done. J. Intern. Med..

[111] Zalewska-Piątek B., Nagórka M., Piątek R. (2025). Role of Uropathogenic *Escherichia coli* and Other Pathogens in Kidney Stone Formation: From Pathogenesis to Treatment. Pathogens.

[112] Karam M.R., Oloomi M., Mahdavi M., Habibi M., Bouzari S. (2013). Assessment of immune responses of the flagellin (FliC) fused to FimH adhesin of Uropathogenic *Escherichia coli*. Mol. Immunol..

[113] Asadi Karam M.R., Habibi M., Bouzari S. (2016). Use of flagellin and cholera toxin as adjuvants in intranasal vaccination of mice to enhance protective immune responses against uropathogenic *Escherichia coli* antigens. Biologicals.

[114] Imani Fooladi A.A., Bagherpour G., Khoramabadi N., Fallah Mehrabadi J., Mahdavi M., Halabian R., Amin M., Izadi Mobarakeh J., Einollahi B. (2014). Cellular immunity survey against urinary tract infection using pVAX/fimH cassette with mammalian and wild type codon usage as a DNA vaccine. Clin. Exp. Vaccine Res..

[115] Saade F., Petrovsky N. (2012). Technologies for enhanced efficacy of DNA vaccines. Expert Rev. Vaccines.

[116] Habibi M., Asadi Karam M.R., Bouzari S. (2015). Evaluation of the effect of MPL and delivery route on immunogenicity and protectivity of different formulations of FimH and MrpH from uropathogenic *Escherichia coli* and Proteus mirabilis in a UTI mouse model. Int. Immunopharmacol..

[117] Asadi Karam M.R., Oloomi M., Mahdavi M., Habibi M., Bouzari S. (2013). Vaccination with recombinant FimH fused with flagellin enhances cellular and humoral immunity against urinary tract infection in mice. Vaccine.

[118] Stins M.F., Prasadarao N.V., Ibric L., Wass C.A., Luckett P., Kim K.S. (1994). Binding characteristics of S fimbriated *Escherichia coli* to isolated brain microvascular endothelial cells. Am. J. Pathol..

[119] Roberts J.A., Kaack M.B., Baskin G., Chapman M.R., Hunstad D.A., Pinkner J.S., Hultgren S.J. (2004). Antibody responses and protection from pyelonephritis following vaccination with purified *Escherichia coli* PapDG protein. J. Urol..

[120] O’Hanley P., Lark D., Falkow S., Schoolnik G. (1985). Molecular basis of *Escherichia coli* colonization of the upper urinary tract in BALB/c mice. Gal-Gal pili immunization prevents *Escherichia coli* pyelonephritis in the BALB/c mouse model of human pyelonephritis. J. Clin. Investig..

[121] Roberts J.A., Hardaway K., Kaack B., Fussell E.N., Baskin G. (1984). Prevention of pyelonephritis by immunization with P-fimbriae. J. Urol..

[122] Savar N.S., Jahanian-Najafabadi A., Mahdavi M., Shokrgozar M.A., Jafari A., Bouzari S. (2014). In silico and in vivo studies of truncated forms of flagellin (FliC) of enteroaggregative *Escherichia coli* fused to FimH from uropathogenic *Escherichia coli* as a vaccine candidate against urinary tract infections. J. Biotechnol..

[123] Valle J., Mabbett A.N., Ulett G.C., Toledo-Arana A., Wecker K., Totsika M., Schembri M.A., Ghigo J.M., Beloin C. (2008). UpaG, a new member of the trimeric autotransporter family of adhesins in uropathogenic *Escherichia coli*. J. Bacteriol..

[124] Durant L., Metais A., Soulama-Mouze C., Genevard J.M., Nassif X., Escaich S. (2007). Identification of candidates for a subunit vaccine against extraintestinal pathogenic *Escherichia coli*. Infect. Immun..

[125] Garcia E.C., Brumbaugh A.R., Mobley H.L. (2011). Redundancy and specificity of *Escherichia coli* iron acquisition systems during urinary tract infection. Infect. Immun..

[126] O’Hanley P., Lalonde G., Ji G. (1991). Alpha-hemolysin contributes to the pathogenicity of piliated digalactoside-binding *Escherichia coli* in the kidney: Efficacy of an alpha-hemolysin vaccine in preventing renal injury in the BALB/c mouse model of pyelonephritis. Infect. Immun..

[127] Moriel D.G., Bertoldi I., Spagnuolo A., Marchi S., Rosini R., Nesta B., Pastorello I., Corea V.A.M., Torricelli G., Cartocci E. (2010). Identification of protective and broadly conserved vaccine antigens from the genome of extraintestinal pathogenic *Escherichia coli*. Proc. Natl. Acad. Sci. USA.

[128] Rippere-Lampe K.E., Lang M., Ceri H., Olson M., Lockman H.A., O’Brien A.D. (2001). Cytotoxic necrotizing factor type 1-positive *Escherichia coli* causes increased inflammation and tissue damage to the prostate in a rat prostatitis model. Infect. Immun..

[129] Smith Y.C., Rasmussen S.B., Grande K.K., Conran R.M., O’Brien A.D. (2008). Hemolysin of uropathogenic *Escherichia coli* evokes extensive shedding of the uroepithelium and hemorrhage in bladder tissue within the first 24 hours after intraurethral inoculation of mice. Infect. Immun..

[130] Smith M.A., Weingarten R.A., Russo L.M., Ventura C.L., O’Brien A.D. (2015). Antibodies against hemolysin and cytotoxic necrotizing factor type 1 (CNF1) reduce bladder inflammation in a mouse model of urinary tract infection with toxigenic uropathogenic *Escherichia coli*. Infect. Immun..

[131] Aziminia N., Hadjipavlou M., Philippou Y., Pandian S.S., Malde S., Hammadeh M.Y. (2019). Vaccines for the prevention of recurrent urinary tract infections: A systematic review. BJU Int..

[132] Nickel J.C., Kelly K.L., Griffin A., Elterman D., Clark-Pereira J., Doiron R.C. (2024). MV140 sublingual vaccine reduces recurrent urinary tract infection in women Results from the first North American clinical experience study. Can. Urol. Assoc. J..

[133] Palladino B., O’Shea-Farren D., Boan P., Ho S., Irish A., Pereira L., Robinson J.O., Swaminathan R. (2025). Safety and Efficacy of MV140 Sublingual Vaccine (Uromune) in Preventing Recurrent Urine Infections Post Renal Transplant: A Case Series. Transpl. Infect. Dis. Off. J. Transplant. Soc..

[134] Billips B.K., Yaggie R.E., Cashy J.P., Schaeffer A.J., Klumpp D.J. (2009). A live-attenuated vaccine for the treatment of urinary tract infection by uropathogenic *Escherichia coli*. J. Infect. Dis..

[135] Ramírez-Sevilla C., Gómez-Lanza E., Llopis-Manzanera J., Cetina-Herrando A., Puyol-Pallàs J.M. (2023). Effectiveness and health cost analysis between immunoprophylaxis with MV140 autovaccine, MV140 vaccine and continuous treatment with antibiotics to prevent recurrent urinary tract infections. Actas Urol. Esp..

[136] Kay E., Cuccui J., Wren B.W. (2019). Recent advances in the production of recombinant glycoconjugate vaccines. npj Vaccines.

[137] Tanabe R.H.S., Dias R.C.B., Orsi H., de Lira D.R.P., Vieira M.A., Dos Santos L.F., Ferreira A.M., Rall V.L.M., Mondelli A.L., Gomes T.A.T. (2022). Characterization of Uropathogenic *Escherichia coli* Reveals Hybrid Isolates of Uropathogenic and Diarrheagenic (UPEC/DEC) *E. coli*. Microorganisms.

[138] Liu B., Furevi A., Perepelov A.V., Guo X., Cao H., Wang Q., Reeves P.R., Knirel Y.A., Wang L., Widmalm G. (2020). Structure and genetics of *Escherichia coli* O antigens. FEMS Microbiol. Rev..

[139] Lacerda Mariano L., Ingersoll M.A. (2020). The immune response to infection in the bladder. Nat. Rev. Urol..

[140] Sun X., Stefanetti G., Berti F., Kasper D.L. (2019). Polysaccharide structure dictates mechanism of adaptive immune response to glycoconjugate vaccines. Proc. Natl. Acad. Sci. USA.

[141] Avci F.Y., Li X., Tsuji M., Kasper D.L. (2011). A mechanism for glycoconjugate vaccine activation of the adaptive immune system and its implications for vaccine design. Nat. Med..

[142] Li J., Ma X., Lin A., Pan H., Hao B., Shao J., Li Y., Xu Y., Shao Z., Xu A. (2025). Development and application of polysaccharide conjugate vaccine carrier protein. Zhonghua Yu Fang Yi Xue Za Zhi Chin. J. Prev. Med..

[143] Arconada Nuin E., Vilken T., Xavier B.B., Doua J., Morrow B., Geurtsen J., Go O., Spiessens B., Sarnecki M., Poolman J. (2024). A microbiological and genomic perspective of globally collected *Escherichia coli* from adults hospitalized with invasive *E. coli* disease. J. Antimicrob. Chemother..

[144] Lipworth S., Vihta K.D., Chau K.K., Kavanagh J., Davies T., George S., Barker L., Vaughan A., Andersson M., Jeffery K. (2021). Ten Years of Population-Level Genomic *Escherichia coli* and *Klebsiella pneumoniae* Serotype Surveillance Informs Vaccine Development for Invasive Infections. Clin. Infect. Dis..

[145] Foroogh N., Rezvan M., Ahmad K., Mahmood S. (2021). Structural and functional characterization of the FimH adhesin of uropathogenic *Escherichia coli* and its novel applications. Microb. Pathog..

[146] Karam M.R.A., Habibi M., Bouzari S. (2019). Urinary tract infection: Pathogenicity, antibiotic resistance and development of effective vaccines against Uropathogenic *Escherichia coli*. Mol. Immunol..

[147] Alteri C.J., Mobley H.L. (2007). Quantitative profile of the uropathogenic *Escherichia coli* outer membrane proteome during growth in human urine. Infect. Immun..

[148] Alteri C.J., Hagan E.C., Sivick K.E., Smith S.N., Mobley H.L. (2009). Mucosal immunization with iron receptor antigens protects against urinary tract infection. PLoS Pathog..

[149] Habibi M., Karam M.R.A., Bouzari S. (2017). Evaluation of prevalence, immunogenicity and efficacy of FyuA iron receptor in uropathogenic *Escherichia coli* isolates as a vaccine target against urinary tract infection. Microb. Pathog..

[150] Mike L.A., Smith S.N., Sumner C.A., Eaton K.A., Mobley H.L. (2016). Siderophore vaccine conjugates protect against uropathogenic *Escherichia coli* urinary tract infection. Proc. Natl. Acad. Sci. USA.

[151] Verez-Bencomo V., Fernández-Santana V., Hardy E., Toledo M.E., Rodríguez M.C., Heynngnezz L., Rodriguez A., Baly A., Herrera L., Izquierdo M. (2004). A synthetic conjugate polysaccharide vaccine against *Haemophilus influenzae* type b. Science.

[152] Gruber W.C., Scott D.A., Emini E.A. (2012). Development and clinical evaluation of Prevnar 13, a 13-valent pneumocococcal CRM197 conjugate vaccine. Ann. N. Y. Acad. Sci..

[153] Croxtall J.D., Keating G.M. (2009). Pneumococcal Polysaccharide Protein D-Conjugate Vaccine (Synflorix™; PHiD-CV). Pediatr. Drugs.

[154] Romano M.R., Berti F., Rappuoli R. (2022). Classical-and bioconjugate vaccines: Comparison of the structural properties and immunological response. Curr. Opin. Immunol..

[155] Frasch C.E. (2009). Preparation of bacterial polysaccharide-protein conjugates: Analytical and manufacturing challenges. Vaccine.

[156] Terra V.S., Mills D.C., Yates L.E., Abouelhadid S., Cuccui J., Wren B.W. (2012). Recent developments in bacterial protein glycan coupling technology and glycoconjugate vaccine design. J. Med. Microbiol..

[157] Wacker M., Feldman M.F., Callewaert N., Kowarik M., Clarke B.R., Pohl N.L., Hernandez M., Vines E.D., Valvano M.A., Whitfield C. (2006). Substrate specificity of bacterial oligosaccharyltransferase suggests a common transfer mechanism for the bacterial and eukaryotic systems. Proc. Natl. Acad. Sci. USA.

[158] Iwashkiw J.A., Vozza N.F., Kinsella R.L., Feldman M.F. (2013). Pour some sugar on it: The expanding world of bacterial protein O-linked glycosylation. Mol. Microbiol..

[159] Kowarik M., Numao S., Feldman M.F., Schulz B.L., Callewaert N., Kiermaier E., Catrein I., Aebi M. (2006). N-linked glycosylation of folded proteins by the bacterial oligosaccharyltransferase. Science.

[160] Kowarik M., Young N.M., Numao S., Schulz B.L., Hug I., Callewaert N., Mills D.C., Watson D.C., Hernandez M., Kelly J.F. (2006). Definition of the bacterial N-glycosylation site consensus sequence. EMBO J..

[161] Faridmoayer A., Fentabil M.A., Mills D.C., Klassen J.S., Feldman M.F. (2007). Functional Characterization of Bacterial Oligosaccharyltransferases Involved in O-Linked Protein Glycosylation. J. Bacteriol..

[162] Pan C., Sun P., Liu B., Liang H., Peng Z., Dong Y., Wang D., Liu X., Wang B., Zeng M. (2016). Biosynthesis of Conjugate Vaccines Using an O-Linked Glycosylation System. mBio.

[163] Wang Y., Wang X., Ma G., Xie L., Liu D., Wang Y., Zhao X., Su Y., Perepelov A.V., Ding P. (2023). Sustainable production of a polysaccharide-based glycoprotein by simultaneous conversion of glucose and glycerol in engineered *Escherichia coli*. Green Chem..

[164] Rappuoli R., De Gregorio E., Costantino P. (2019). On the mechanisms of conjugate vaccines. Proc. Natl. Acad. Sci. USA.

[165] Feldman M.F., Wacker M., Hernandez M., Hitchen P.G., Marolda C.L., Kowarik M., Morris H.R., Dell A., Valvano M.A., Aebi M. (2005). Engineering N-linked protein glycosylation with diverse O antigen lipopolysaccharide structures in *Escherichia coli*. Proc. Natl. Acad. Sci. USA.

[166] Peng Z., Wu J., Wang K., Li X., Sun P., Zhang L., Huang J., Liu Y., Hua X., Yu Y. (2021). Production of a Promising Biosynthetic Self-Assembled Nanoconjugate Vaccine against Klebsiella Pneumoniae Serotype O2 in a General *Escherichia Coli* Host. Adv. Sci..

[167] Feldman M.F., Mayer Bridwell A.E., Scott N.E., Vinogradov E., McKee S.R., Chavez S.M., Twentyman J., Stallings C.L., Rosen D.A., Harding C.M. (2019). A promising bioconjugate vaccine against hypervirulent Klebsiella pneumoniae. Proc. Natl. Acad. Sci. USA.

[168] Hao L., Huang W., Guo Y., Liu X., Wu J., Zhu L., Pan C., Wang H. (2025). A Bioconjugate Vaccine Against Extra-Intestinal Pathogenic *Escherichia coli* (ExPEC). Vaccines.

[169] Pan C., Wu J., Qing S., Zhang X., Zhang L., Yue H., Zeng M., Wang B., Yuan Z., Qiu Y. (2020). Biosynthesis of Self-Assembled Proteinaceous Nanoparticles for Vaccination. Adv. Mater..

[170] Jiang X., Bai J., Zhang H., Yuan J., Lu G., Wang Y., Jiang L., Liu B., Huang D., Feng L. (2022). Development of an O-polysaccharide based recombinant glycoconjugate vaccine in engineered *E. coli* against ExPEC O1. Carbohydr. Polym..

[171] Jiang X., Bai J., Yuan J., Zhang H., Lu G., Wang Y., Jiang L., Liu B., Wang L., Huang D. (2021). High efficiency biosynthesis of O-polysaccharide-based vaccines against extraintestinal pathogenic *Escherichia coli*. Carbohydr. Polym..

[172] Wang Y., Perepelov A.V., Senchenkova S.N., Lu G., Wang X., Ma G., Yang Q., Yuan J., Wang Y., Xie L. (2023). Glycoengineering directs de novo biomanufacturing of UPEC O21 O-antigen polysaccharide based glycoprotein. Int. J. Biol. Macromol..

[173] Watt J.P., Levine O.S., Santosham M. (2003). Global reduction of Hib disease: What are the next steps? Proceedings of the meeting: Scottsdale, Arizona, September 22–25, 2002. J. Pediatr..

[174] Jefferies J.M., Macdonald E., Faust S.N., Clarke S.C. (2011). 13-valent pneumococcal conjugate vaccine (PCV13). Hum. Vaccines.

[175] Dagan R. (2009). Serotype replacement in perspective. Vaccine.

[176] Hsu H.E., Shutt K.A., Moore M.R., Beall B.W., Bennett N.M., Craig A.S., Farley M.M., Jorgensen J.H., Lexau C.A., Petit S. (2009). Effect of pneumococcal conjugate vaccine on pneumococcal meningitis. N. Engl. J. Med..

[177] Sze C., Attia S., Zimmern P. (2025). Effective risk reduction strategies and pharmacological treatment for uncomplicated recurrent urinary tract infections. Expert Opin. Pharmacother..

[178] Schiøtz H.A., Guttu K. (2002). Value of urinary prophylaxis with methenamine in gynecologic surgery. Acta Obstet. Gynecol. Scand..

[179] Carmain M., Sappenfield E.C., Tunitsky-Bitton E. (2025). Probiotics: Utility, benefits, and risks for gynecologic conditions. Curr. Opin. Obstet. Gynecol..

[180] Chaudhary N., Kaur H., Modgil V., Singh D., Maurya R.K., Mohan B., Taneja N. (2025). In vitro and In vivo evaluation of phage-antibiotic synergy for the treatment of urinary tract infections. Microb. Pathog..

[181] Siddiqui N.Y., Bradley M.S. (2022). Updates in clinical management of recurrent urinary tract infections. Obstet. Gynecol..

[182] Foxman B., Cronenwett A.E., Spino C., Berger M.B., Morgan D.M. (2015). Cranberry juice capsules and urinary tract infection after surgery: Results of a randomized trial. Am. J. Obstet. Gynecol..

[183] Kostiala A.A., Nyrén P., Runeberg L. (1982). Effect of nitrofurantoin and methenamine hippurate prophylaxis on bacteria and yeasts in the urine of patients with an indwelling catheter. J. Hosp. Infect..

[184] Norrman K., Wibell L. (1976). Treatment with methenamine hippurate† in the patient with a catheter. J. Int. Med. Res..

[185] Murali Krishna M., Joseph M., Pereira V., Nizami A., Ezenna C., Sadasivan Sreemathy L. (2024). D-Mannose for prevention of recurrent urinary tract infection in adult women: An updated systematic review and meta-analysis of randomized controlled trials. J. Infect. Prev..

[186] Bossa L., Kline K., McDougald D., Lee B.B., Rice S.A. (2017). Urinary catheter-associated microbiota change in accordance with treatment and infection status. PLoS ONE.

[187] Saz-Leal P., Ligon M.M., Diez-Rivero C.M., García-Ayuso D., Mohanty S., Viñuela M., Real-Arévalo I., Conejero L., Brauner A., Subiza J.L. (2024). MV140 mucosal vaccine induces targeted immune response for enhanced clearance of uropathogenic *E. coli* in experimental urinary tract infection. Vaccines.

[188] Pichichero M.E. (2013). Protein carriers of conjugate vaccines: Characteristics, development, and clinical trials. Hum. Vaccines Immunother..

[189] Peeters C.C., Tenbergen-Meekes A.M., Poolman J.T., Beurret M., Zegers B.J., Rijkers G.T. (1991). Effect of carrier priming on immunogenicity of saccharide-protein conjugate vaccines. Infect. Immun..

[190] Dagan R., Eskola J., Leclerc C., Leroy O. (1998). Reduced response to multiple vaccines sharing common protein epitopes that are administered simultaneously to infants. Infect. Immun..

[191] Cuccui J., Thomas R.M., Moule M.G., D’Elia R.V., Laws T.R., Mills D.C., Williamson D., Atkins T.P., Prior J.L., Wren B.W. (2013). Exploitation of bacterial N-linked glycosylation to develop a novel recombinant glycoconjugate vaccine against *Francisella tularensis*. Open Biol..

[192] Ma Z., Zhang H., Shang W., Zhu F., Han W., Zhao X., Han D., Wang P.G., Chen M. (2014). Glycoconjugate vaccine containing *Escherichia coli* O157:H7 O-antigen linked with maltose-binding protein elicits humoral and cellular responses. PLoS ONE.

[193] Smith D.M., Simon J.K., Baker J.R. (2013). Applications of nanotechnology for immunology. Nat. Rev. Immunol..

